# Effects of set cathode potentials on microbial electrosynthesis system performance and biocathode methanogen function at a metatranscriptional level

**DOI:** 10.1038/s41598-020-76229-5

**Published:** 2020-11-13

**Authors:** Ala’a Ragab, Dario Rangel Shaw, Krishna P. Katuri, Pascal E. Saikaly

**Affiliations:** Biological and Environmental Science and Engineering Division, Water Desalination and Reuse Center, King Abdullah University of Science and Technology (KAUST), Thuwal, Saudi Arabia

**Keywords:** Applied microbiology, Archaea, Bacteria, Biofilms, Environmental microbiology, Microbial genetics, Environmental sciences

## Abstract

Microbial electrosynthesis exploits the catalytic activity of microorganisms to utilize a cathode as an electron donor for reducing waste CO_2_ to valuable fuels and chemicals. Electromethanogenesis is the process of CO_2_ reduction to CH_4_ catalyzed by methanogens using the cathode directly as a source of electrons or indirectly via H_2_. Understanding the effects of different set cathode potentials on the functional dynamics of electromethanogenic communities is crucial for the rational design of cathode materials. Replicate enriched electromethanogenic communities were subjected to different potentials (− 1.0 V and − 0.7 V vs. Ag/AgCl) and the potential-induced changes were analyzed using a metagenomic and metatranscriptomic approach. The most abundant and transcriptionally active organism on the biocathodes was a novel species of *Methanobacterium* sp. strain 34x. The cathode potential-induced changes limited electron donor availability and negatively affected the overall performance of the reactors in terms of CH_4_ production. Although high expression of key genes within the methane and carbon metabolism pathways was evident, there was no significant difference in transcriptional response to the different set potentials. The acetyl-CoA decarbonylase/synthase (ACDS) complex were the most highly expressed genes, highlighting the significance of carbon assimilation under limited electron donor conditions and its link to the methanogenesis pathway.

## Introduction

Microbial electrochemical technologies (METs) exploit the catalytic activity of microorganisms for electric current generation and different chemical product formation. The main explored application for METs in the last two decades has been wastewater treatment (organic oxidation and removal) with simultaneous energy recovery in the form of electricity (microbial fuel cell; MFC)^1^ or H_2_ (microbial electrolysis cell; MEC)^2–5^. These applications have been expanded to include reduction of CO_2_ waste streams to CH_4_, different volatile fatty acids and alcohols at the cathode of microbial electrosynthesis (MES) systems^6–11^. MES is a promising application that can be utilized in biogas upgrading and renewable energy storage^12^. Electromethanogenesis in MES systems is the process of CO_2_ reduction to CH_4_, a process that is catalyzed by methanogens using a cathode as the electron donor directly (Eq. 1), or indirectly via H_2_, produced through the hydrogen evolution reaction (HER) (Eqs. 2 and 3)^8,13,14^.

1 $$ {\text{CO}}_{{2}} + {\text{8e}}^{ - } + {\text{ 8H}}^{ + } \to {\text{CH}}_{{4}} + {\text{2H}}_{{2}} {\text{O}} \;\,\;\; - 0.{443}\;{\text{V}}\;{\text{vs}}.\;{\text{Ag}}/{\text{AgCl}},\;{\text{ pH}}\;{7} $$

2 $$ {\text{8H}}^{ + } + {\text{8e}}^{ - } \to {\text{4H}}_{{2}} \;\;\; - 0.{613}\;{\text{V}}\;{\text{vs}}.\;{\text{Ag}}/{\text{AgCl}},\;\;{\text{pH}}\;{7} $$

3 $$ {\text{CO}}_{{2}} + {\text{4H}}_{{2}} \to {\text{CH}}_{{4}} + {\text{ 2H}}_{{2}} {\text{O}}\;\;\;\Delta G = - {131}\;{\text{kJ}}/{\text{mol}},\;{\text{ pH}}\;{ 7} $$

Direct electron transfer encompasses the transfer of free electrons from cell to a solid surface (i.e. the anode in MFC or MEC) and vice versa (i.e. the cathode in MES or MEC), as well as the transfer of electrons between species through direct connections or electrically conductive materials^15^. Although it is more energetically favorable to produce CH_4_ via direct electron transfer, this process is limited by the need for direct contact between the cathode and microbe. This specific area imitation requires 3D architecture- cathodes to increase the area available for attachment and overall reaction kinetics.

Many MES studies have shown the dominance of H_2_-mediated electron transfer^12,13,16–19^. Indirect electron transfer rates are highly dependent on substrate concentration gradients within the cathodic biofilm and are regulated by the energy available from microbial catabolic reactions^20,21^ and the HER efficiency of the cathode material^15^.

Three pathways are known for CH_4_ production by methanogens: hydrogenotrophic, acetoclastic and methylotrophic methanogenesis. Hydrogenotrophic methanogens such as *Methanobacterium* spp. are regularly described as dominating methanogenic biocathodes, with H_2_ generally as the electron donor and CO_2_ as the terminal electron acceptor (Eqs. 2 and 3)^12,22^. H_2_ scavenging by other microbes can also occur since H_2_ is a ubiquitous electron donor^23^. Acetate, which is activated into acetyl-CoA by the reductive acetyl-CoA pathway, is the terminal electron acceptor in acetoclastic methanogenesis. The reductive acetyl-CoA pathway is also crucial for carbon assimilation in all methanogens. Although MES refers to CO_2_ capture and synthesis into value-added products using the cathode as an electron donor, acetate can arise in these systems if homoacetogens are present or due to cell decay in mixed culture systems. Therefore, acetoclastic methanogens such as *Methanosarcina* spp. are also frequently reported in biocathodes^12,22^.

Since the cathode as the electron donor is critical to control reaction kinetics, CH_4_ production rates, and yields, it is important to focus on the cathode to further develop MES systems for commercial scale-up^15^. Cathodic reactions vary in their theoretical onset potentials due to thermodynamic differences^16^. Therefore, set cathode potential is a crucial parameter to optimize in MES systems for CO_2_ reduction to CH_4_. Several studies have explored the effect of the set cathode potential in MES on reactor performance metabolic pathways, demonstrating that lower potentials lead to improved production rates^8,13,24,25^. However, there remains a knowledge gap regarding the functional dynamics of methanogenic MES biocathode performance in response to different cathode potentials. Although there are reports investigating transcriptional changes in methanogenic MES biocathode communities in response to set cathode potential compared to open circuit conditions^26^, to our knowledge, there are no studies that evaluate the functional response to different set potentials of methanogenic biocathodic communities at a metatranscriptional level.

Differences in set cathode potentials can affect hydrogen and electron availability at the cathode, hence affecting CH_4_ metabolism-related gene expression. Studying the transcriptional changes under different set cathode potentials may highlight central genes in electron transfer for CO_2_ reduction to CH_4_. Based on the Reactions (1) and (2) outlined above, at less negative cathode set potentials (− 0.443 V vs. Ag/AgCl), abiotic hydrogen evolution should be minimal due to thermodynamic limitations with HER; thus, theoretically CO_2_ reduction should take place according to Reaction (1) with electrons directly transferred from cathode to methanogens. This would limit indirect H_2_-mediated electron transfer and lead to transcriptional changes in the genes involved in direct electron transfer. At more negative cathode set potentials (≥ − 0.613 V vs. Ag/AgCl), more abiotic hydrogen will evolve and a combination of direct and indirect electron transfer would take place depending on the microbial community response. Therefore, the objective of this study was to understand the effect of set cathode potential on MES reactor performance and functional dynamics of biocathode methanogenic community using a genome-centric metatranscriptomics approach. The knowledge gained from this work aids in understanding the metabolic responses of methanogenic microbial communities to different operational conditions. This furthers work towards microbial-driven process control in electromethanogenic systems. Understanding the metabolic responses of methanogenic microbial communities to different operational conditions will aid in improving MES systems’ performance for practical applications^15^.

## Results

### MES performance before set potential changes

The biocathodes were enriched for methanogens in single-chamber reactors operated for 5 months in MEC mode, with an applied voltage of 0.7 V and the presence of acetate as a carbon source. The average recorded current density for the triplicate reactors was 0.045 ± 0.005 mA/cm^2^ (s.d.), the average r_catCH4_ was 155.0 ± 80.9% (s.d.), and the coulombic efficiency (CE) was 74.4 ± 9% (s.d.). The presence of CH_4_ indicated the presence of a methanogenic community, and thus the enriched biocathodes were transferred to double-chambered reactors for MES operation as reactors R1, R2 and R3. These were operated at a set cathode potential of − 1.0 V (vs. Ag/AgCl) and batch-fed with CO_2_ (gas and bicarbonate) as the only externally added carbon source. The reactors were operated under these conditions for seven batches (140 h batch-length), until stable CH_4_ production was observed for three consecutive batches (ANOVA, F = 4.2, p > 0.05). The MES cathodic current density was between − 0.02 and − 0.05 mA/cm^2^, with an overall increase in current consumption over time (Supplementary Fig. S1). Under MES operation, CH_4_ concentration varied significantly between batches, averaging 14.7 ± 10.5 µmol /cm^2^ (Kruskal–Wallis, χ = 7.7, p = 0.005). However, in considering the conversion efficiency of the total coulombs available at the cathode for CO_2_ reduction to CH_4_ (r_catCH4_), the CH_4_ production did not vary significantly (ANOVA, F = 4.2, p > 0.05). This was also true for H_2_ production, where the H_2_ concentration significantly varied (Kruskal–Wallis, χ = 4.3, p = 0.04), while the conversion efficiency to H_2_ (r_catH2_) did not (ANOVA, F = 1.8, p > 0.05). The average recorded HER rate was 0.03 ± 0.01 µmol/cm^2^/h (s.d.), compared to an abiotic HER rate of 6.3 ± 1.5 µmol/cm^2^/h (s.d.). The biotic HER rate was lower than the abiotic rate due to microbial consumption of H_2_. Both electrode-assisted methanogenesis and acidogenesis occurred, as evidenced by the products detected at the end of each batch (CH_4_, formate and acetate). The VFA production was not significantly variable between batches (p > 0.05). Formate concentrations varied between undetectable levels up to a maximum of 2.5 µmol/cm^2^, averaging 0.6 µmol/cm^2^ ± 1.0 (s.d.) overall (ANOVA, p > 0.05). Acetate levels varied between batches, with an average concentration of 5.5 ± 0.07 µmol/cm^2^ (s.d.) (Kruskal–Wallis, p > 0.05).

### Current density and gas performance in response to set potential changes

Both current density and gas production were adversely affected by decreasing the cathode potential from − 1.0 V to − 0.7 V for the three reactors (Table 1). This was expected, as less negative cathode potential reduces electron availability along the cathode as charge and H_2_ via HER. The biocathodes responded quickly to potential-induced changes; H_2_ and CH_4_ reached detectable limits within 45 min after switching the cathode potential from − 1.0 V to − 0.7 V. Although no VFAs were detected during the baseline − 1.0 V sampling, acetate and formate concentrations were much higher than the gas products, with formate at 0.6–0.7 µmol/cm^2^ and acetate reaching up to 2.6 µmol/cm^2^. However, after prolonged operation at − 0.7 V (90 min), the gas products and formate were below detectable limits while acetate concentrations were greatly increased to > 10 µmol/cm^2^ for reactors R1 and R2, and 3.5 µmol/cm^2^ in reactor R3.

**Table 1:** Current density j (mA/cm^2^) and product concentration (µmol/cm^2^) recorded for each reactor.

Parameter	− 1.0 V, baseline			− 0.7 V, 45 min			− 0.7 V, 90 min		
	R1	R2	R3	R1	R2	R3	R1	R2	R3
*j*	0.008	0.007	0.05	5.9 × 10^–6^	6.9 × 10^–4^	4.6 × 10^–4^	1.0 × 10^–4^	7.8 × 10^–4^	1.1 × 10^–3^
H_2_	0.47	0.28	0.02	0.03	0.08	0.002	0	0	0
CH_4_	0.05	0.10	0.03	0.08	0.08	0	0	0	0
Formate	0	0	0	0.67	0.64	0.59	0	0	0
Acetate	0	0	0	0.69	0	2.55	10.73	10.08	3.48

The “ − 1.0 V, baseline” refers to performance during the 1-h recovery after the baseline biofilm sampling that preceded the change in set cathode potential experiment.

### Methanogenic core dominant community

The cathodic microbial communities were analyzed using 16S rRNA gene amplicon sequencing of the − 1.0 V baseline biofilm samples. Sequence reads for the replicates ranged between 47,369 and 51,415. After quality filtering, the total 150,141 reads were resolved into 145 observed OTUs. The sampling depth was sufficient to capture most of the species in the samples, as shown in the rarefaction curves (Supplementary Fig. S2). Sample diversity was calculated based on observed OTUs, Shannon–Weaver, Simpson’s Diversity and Chao1 richness estimator after rarefying to 47,369 reads (Supplementary Table S1). Overall, the species richness and diversity was similar amongst the replicates, although reactor R3 had the lowest alpha diversity for all indices.

**Figure 1:**
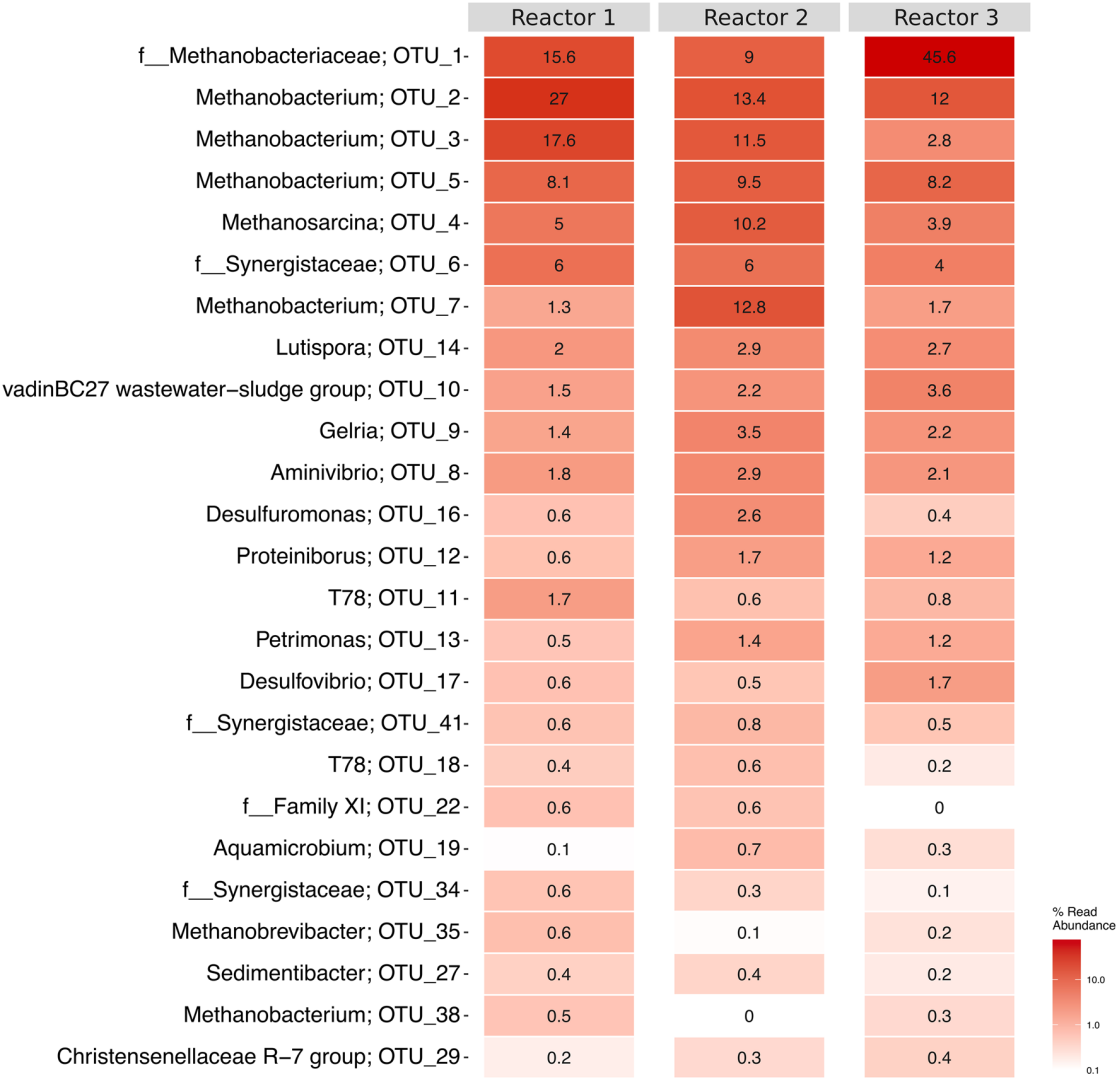
Heatmap of the relative abundance of the 25 OTUs associated with the core dominant community (≥ 0.1%) at the genus or family (f) level with the corresponding specific OTU.

The core dominant community is defined as the OTUs present in all samples at a relative abundance ≥ 0.1%, representing ≥ 80% of total reads^27^. 25 OTUs represented the core dominant community with > 95% of the total reads in the three baseline samples, indicating highly enriched and highly similar microbial communities amongst the three reactors (Supplementary Fig. S3). The relative abundances and taxonomic classification of these 25 OTUs are shown in a heatmap (Fig. 1). The biocathodes were dominated by methanogenic communities, mainly hydrogenotrophic *Methanobacterium* sp. (5 OTUs), as well as *Methanobrevibacter* sp. (1 OTU) and the mixotrophic *Methanosarcina* sp. (1 OTU). Additionally, sulfate-reducing bacteria (SRB) of the genus *Desulfovibrio* and various fermentative bacteria of the phyla Bacteroidetes, Synergistetes, Firmicutes and Chloroflexi (such as *Aminivibrio, Gelria, Lutispora, Petrimonas, Proteiniborus* sp.) were also represented in the core community.

The remaining non-core OTUs (< 0.1% relative abundance in the triplicate samples) also included fermentative bacteria as well as one OTU corresponding to *Acetoanaerobium* sp. There have been reports of H_2_-dependent acetate production by *Acetoanaerobium noterae*^28^, although this OTU was present in lower abundance (0.2% in R1, 0.4% in R2 and 0.04% in R3) (Supplementary Table S2).

### Metagenomic analysis reveals the dominance of a novel Methanobacterium sp.

**Figure 2:**
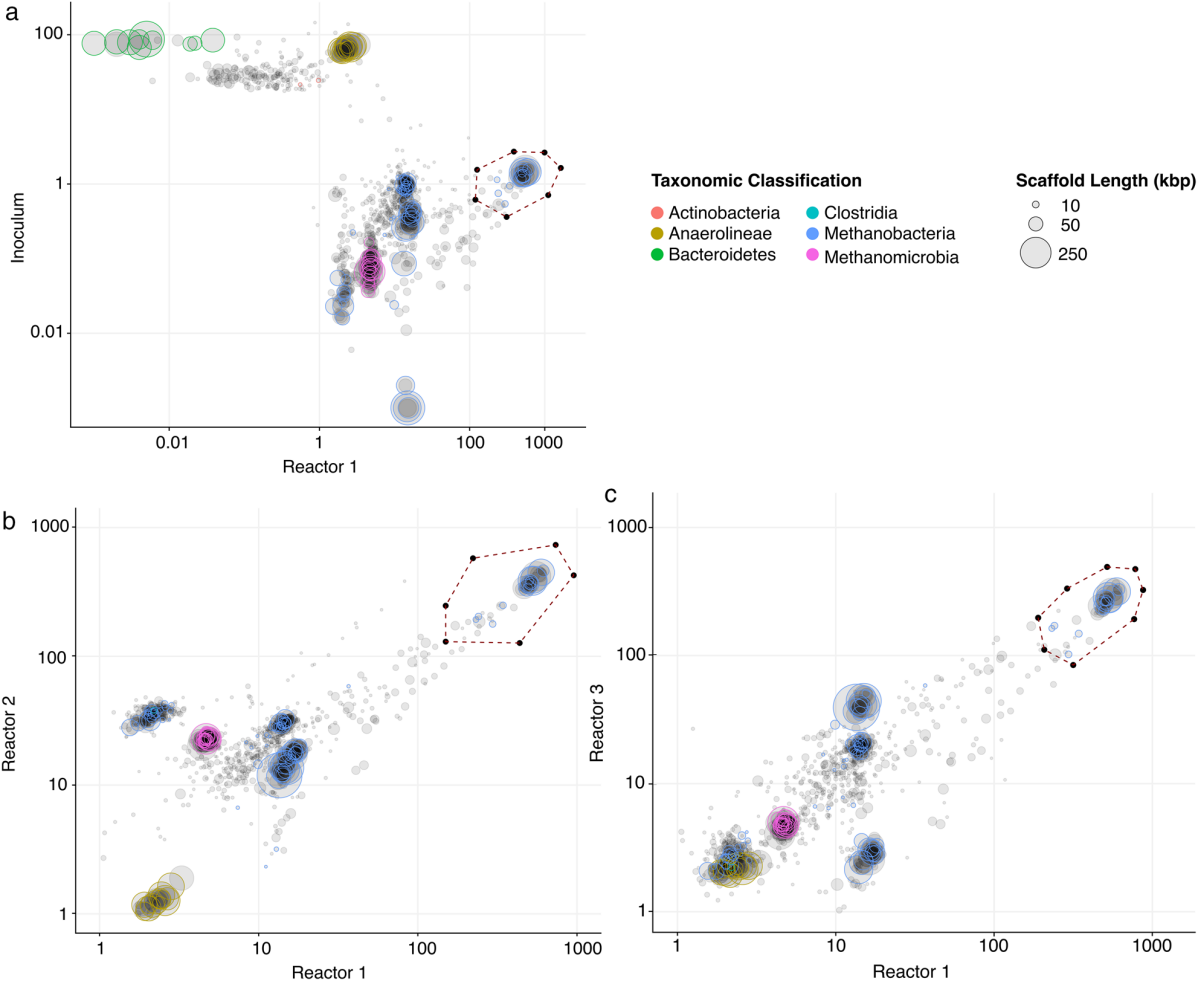
Differential coverage plot of the assembled metagenomic scaffolds (> 10,000 bp) from the − 1.0 V baseline samples taken from each reactor and the initial inoculum, highlighting the extraction of the scaffolds for Bin 1. The size of the circles represents the length of the scaffolds. The colors of the circles represent a phylum-level taxonomic classification based on the essential genes identified in the scaffolds. Scaffolds in grey either contain no essential genes or could not be assigned a phylum-level classification. The x and y-axes show the log-scaled sequencing coverage in the samples. **a** Shows the comparison of coverage plots of Reactor 1 to “a”, the initial inoculum; **b**, Reactor 2; and “c”, Reactor 3.

The most abundant bin amongst all the reactors was Bin 1, corresponding to a *Methanobacterium* species. This is shown in the differential coverage plot of the assembled metagenomic scaffolds (Fig. 2). This bin was highly enriched in the reactors in comparison to the initial inoculum (Fig. 2a), with > 97% similarity, based on the BLASTn results, to *Methanobacterium* OTU_2 and OTU_5 retrieved through Amplicon sequencing of the 16S rRNA genes.

**Figure 3:**
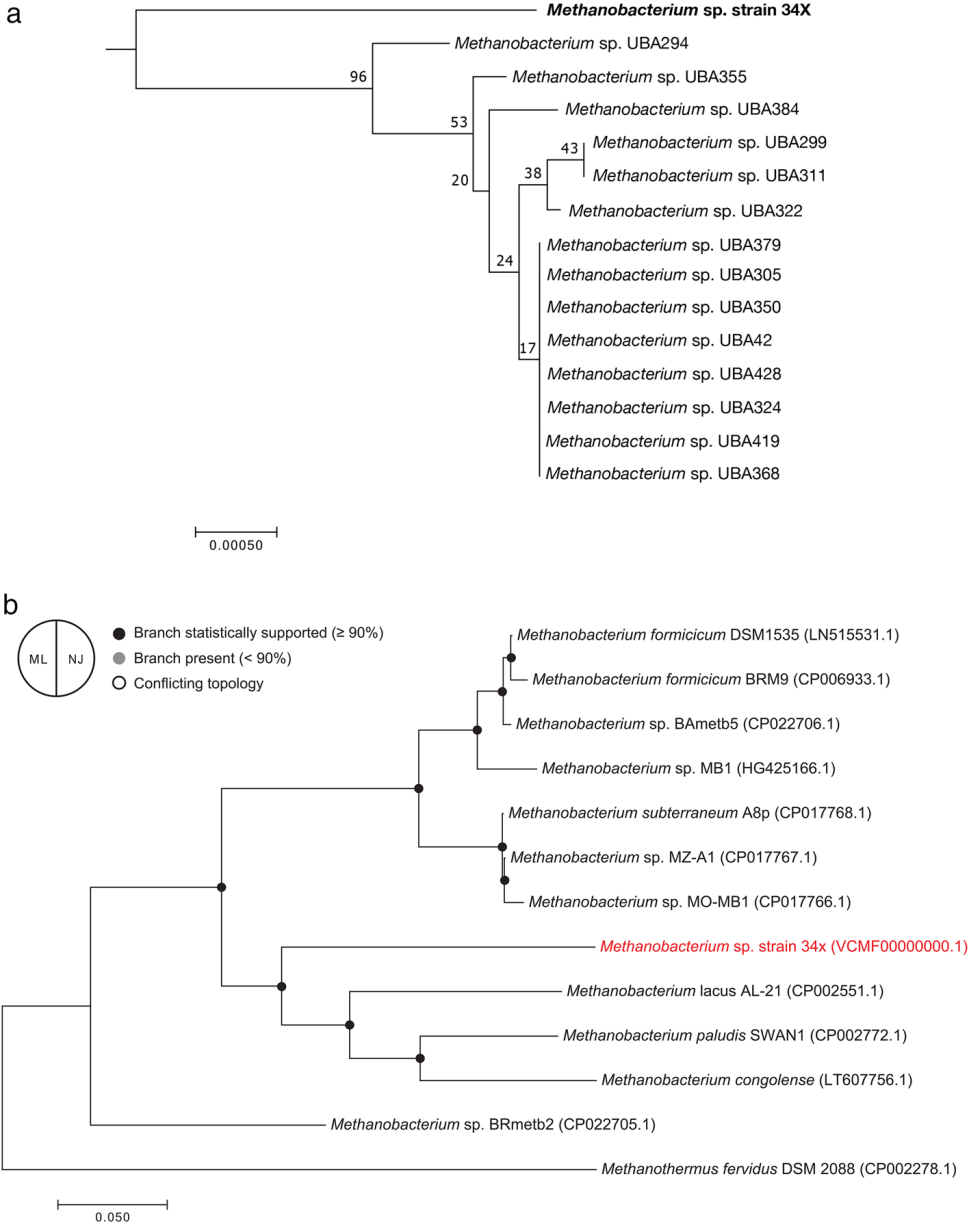
Evolutionary relationships of taxa using phylogenetic trees generated by the Neighbor-Joining (N-J) method (**a**) and the Maximum Likelihood (ML) method (**b**). The N-J tree was generated using all 90 *Methanobacterium* sequences available in the NCBI database; only a zoomed in version of the tree is shown here for clarity. The ML tree was generated using representative sequences commonly used in the literature. The percentage of replicate trees in which the associated taxa clustered together in the bootstrap test (1000 replicates) are shown next to the branches^30^. The trees are drawn to scale, with branch lengths in the same units as those of the evolutionary distances used to infer the phylogenetic trees. The evolutionary distances were computed using the Poisson correction method^31^ and are in the units of the number of amino acid substitutions per site. Fewer than 5% alignment gaps, missing data and ambiguous bases were allowed at any position; all positions with less than 95% site coverage were eliminated. Evolutionary analyses were conducted in MEGA7^32^.

The extracted Bin 1 genome had a size of 2.2 Mbp, with a mean GC content of 37% (Supplementary Table S3). The percentage of metagenomic reads mapped to Bin 1 was between 68 and 83% of the total reads in the triplicate reactors (Supplementary Fig. S4). Average Nucleotide Identity by Orthology (OrthoANI) calculations (Supplementary Table S4) showed that the extracted Bin 1 has less than 70% similarity compared to representative whole-genome assemblies of *Methanobacterium* spp., suggesting that Bin 1 is a novel *Methanobacterium* species. This was further confirmed by phylogenomic analyses comparing the extracted Bin 1 to 90 available *Methanobacterium* sequences in the NCBI database, where there was a clear evolutionary distance between Bin 1 and the most closely related sequences reported to date (Fig. 3, analysis done June, 2019). Therefore, the extracted Bin 1 was designated *Methanobacterium* sp. strain 34x^29^.

Of the 2141 genes identified and functionally annotated with the PROKKA, and COG databases, 457 were annotated as “hypothetical proteins”, 1008 had an “unknown function” or only a “general function prediction’. The remaining 676 genes were involved in a variety of different functions, including energy production and conversion (81 genes), transcription (18 genes), translation (90 genes), replication (33 genes), cell cycle control (12 genes) and cell wall biogenesis (43 genes). Other metabolic-related genes included those involved in coenzyme transport and metabolism (74 genes), amino acid metabolism (87 genes), carbohydrate metabolism (40 genes), lipid metabolism (23 genes) and nucleotide metabolism (40 genes) (Supplementary Table S5).

### Metatranscriptomic analysis

Transcriptomic analyses were focused on the extracted Bin 1 since it was the most abundant bin in all reactors (Fig. 2 and Supplementary Fig. S5) and no less than 78% of the non-RNA reads were mapped to it (Supplementary Table S6 and Fig. S5). Of the total 2,141 genes expressed, there was no significant differential expression (*p* > 0.05) between the − 1.0 V baseline vs. − 0.7 V (45 min), − 1.0 V baseline vs. − 0.7 V (90 min), and between − 0.7 V (45 min) vs. − 0.7 V (90 min) (Supplementary Fig. S6). Pairwise comparisons of the number of reads per gene per sample showed that both intra- and inter-replication was very high (> 90% Pearson’s correlation) in all the samples for all the conditions. These results suggested that Bin 1 was possibly using the same pathway(s) under the two different potentials, or that the change in stimulus-induced potential growth was not sufficient to produce a significant change in expression profile. Comparing the expression levels of genes (transcripts per million, TPM) involved in CH_4_ metabolism and carbon fixation further supported this (Supplementary Table S7). TPM is a unitless normalization measure of relative abundance of mapped reads for any one gene transcript in a sample. Although care must be taken when comparing relative abundance across samples, TPM is a widely accepted normalization method as it respects the invariance property of the average RNA abundance over genes in proportion to the relative RNA molar concentration within a sample^33^.

**Figure 4:**
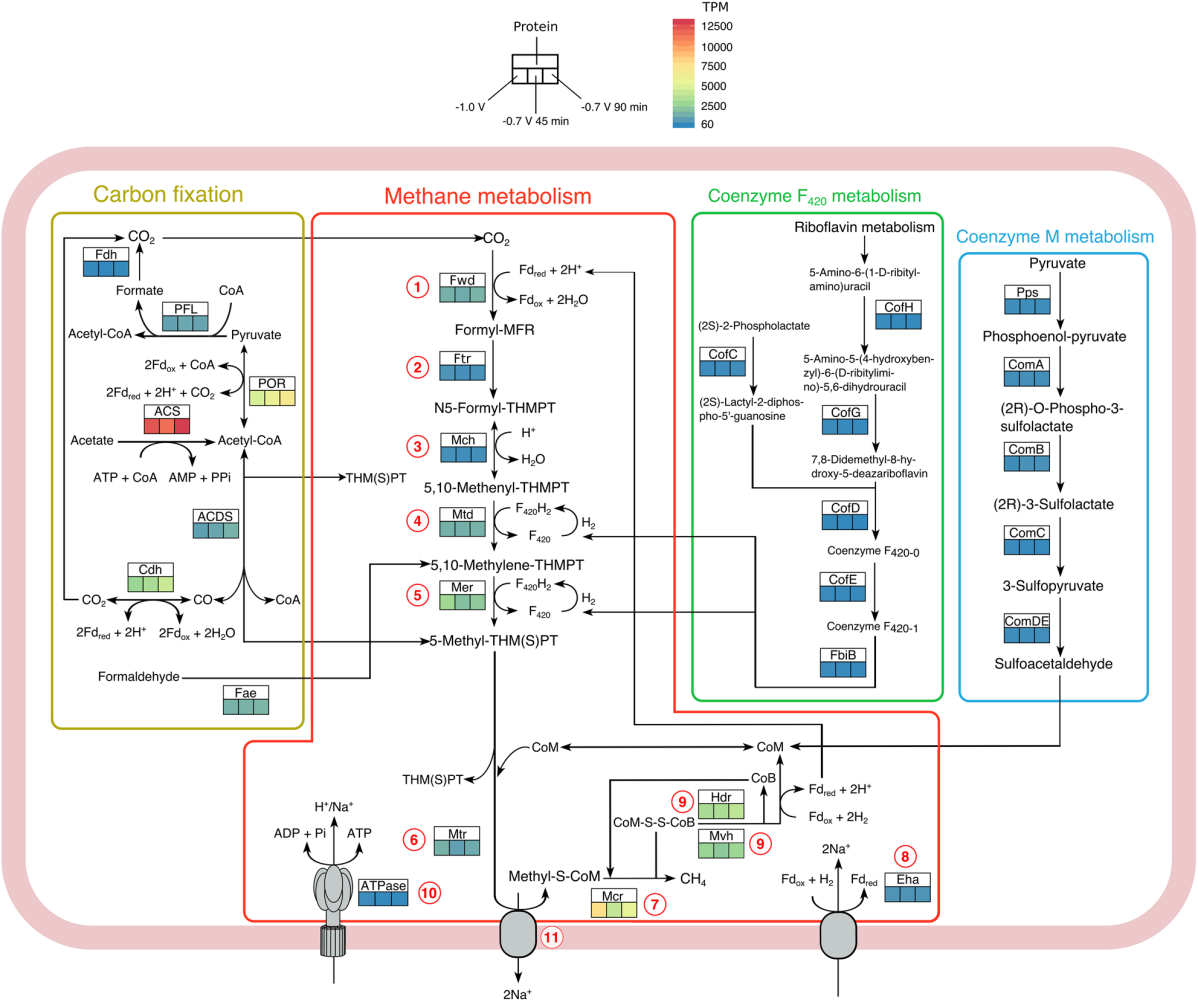
Reconstructed hydrogenotrophic methanogenesis metabolic pathway with associated carbon fixation through the reductive Acetyl-CoA pathway, methane metabolism and coenzyme biosynthesis by *Methanobacterium* sp. strain 34x. Supplementary Table S7 details the genes and enzymes associated with each protein shown in the model. The average expression for the three conditions is color coded in transcripts per million (TPM). The red numbering denotes different steps in the hydrogenotrophic methanogenesis pathway, detailed in Supplementary Table S8. Fd_ox_, oxidized ferredoxin; Fd_red_, reduced ferredoxin; F_420_, coenzyme F_420_; *MFR* methanofuran; *THMPT* tetrahydromethanopterin.

The expression level results (Supplementary Table S7) were used to reconstruct a metabolic pathway for *Methanobacterium* Bin 1 (Fig. 4). The reconstructed metabolic pathway highlights the increased expression levels of specific genes under potential-induced growth. Within the CH_4_ metabolism pathway, which is a 7-step process^34^ outlined in Fig. 4 and Table S7, higher expression of the genes for enzymes catalyzing the reduction of the intermediate methylene- H_4_MPT to CH_4_ was observed (Reactions 4–7, Supplementary Table S8). These include F_420_-dependent methylene- H_4_MPT dehydrogenase, Mtd (Reactions 4), F420-dependent methylene- H_4_MPT reductase, Mer (Reaction 5), Methyl- H_4_MPT:coenzyme M methyltransferase, Mtr (Reaction 6) and methyl-coenzyme M reductase; Mcr (Reaction 7). While no significant differential was observed between the different samples, the basal Mcr expression at − 1.0 V was relatively higher than after the switch to − 0.7 V. The hydrogenase:heterodisulfide reductase (Mvh:Hdr) complex (Reactions 9 and 10 was also highly expressed under the different test conditions (Fig. 4 and Supplementary Table S8). The Hdr complex is crucial for electron bifurcation, a process that links the exergonic heterodisulfide reduction with ferredoxin reduction in Reaction 9 to CO_2_ reduction to formyl-methanofuran by Fwd in Reaction 1^34–37^. The expression of the co-enzyme F_420_ and M pathways associated with methanogenesis was maintained at low basal expression levels throughout. These co-enzymes can be regenerated through H_2_ and would not need to be continuously expressed^38^, therefore low expression levels can be expected. Collectively, these results suggest that the methanogenesis pathway was active throughout the experiment, despite the drop in detectable CH_4_ after switching cathode potential from − 1.0 V to − 0.7 V.

Carbon fixation genes were also highly expressed, with acetyl-coenzyme A synthetase (ACS) as the most expressed gene (12,079- 13,349 TPM) of the total mapped genes in the replicates under all conditions (Fig. 4 and Supplementary Table S7). ACS is part of the ACDS complex (acetyl-CoA decarbonylase/synthase complex), which is involved in carbon assimilation. The ACDS complex activates acetate into acetyl-CoA via the reductive acetyl-CoA pathway with < 1 mol ATP input. Acetyl-CoA can be converted to pyruvate via pyruvate ferredoxin oxidoreductase (Por), which was also highly expressed, and utilized in the pyruvate metabolic pathway for biogenesis.

## Discussion

Many MET systems report the biocathodic enrichment of hydrogenotrophic methanogens^12,23,39^, with *Methanobacterium* sp. being frequently described. This study presents the novel *Methanobacterium* sp. strain 34 × which was selectively enriched on triplicate biocathodes from a diverse inoculum. In hydrogenotrophic methanogenesis, the electron donor is generally recognized to be H_2_ and formate to a lesser degree, although H_2_-independent mechanisms have been reported^40–43^.

### Potential-induced dynamics of product formation and consumption in enriched microbial communities

H_2_ evolves abiotically from the cathode as a function of the set cathode potential within electromethanogenic systems. With a theoretical HER onset potential of − 0.6 V vs. Ag/AgCl, more negative potentials yield higher HER while less negative potentials limit the abiotic H_2_ availability. This would be expected to affect the expression of methanogenesis-related genes in hydrogenotrophic methanogens. Both hydrogen-mediated and direct electromethanogenesis are expected to occur at − 1.0 V vs. Ag/AgCl using carbon cloth^12,17,26,44^ in a double-chambered MES fed with CO_2_, where systems operated at more negative cathode potentials report increased current consumption and CH_4_ production rates (Supplementary Table S9). This holds true for our results as well when comparing the rates at the more negative potential of − 1.0 V compared to − 0.7 V. Based on the gas production observed at − 1.0 V compared to − 0.7 V, 45 and 90 min of growth, H_2_ was clearly limited by the set potential change and therefore CH_4_ production as well.

While the change in set cathode potential negatively impacted current consumption and gas production, an increase in VFA (formate and acetate) concentration was observed upon switching from − 1.0 V to − 0.7 V. The increase in formate may have been due to H_2_ limitation, where formate acted as the electron donor as discussed further below. It is not clear why acetate concentration increased with the higher cathode potential. Acetate may have been produced by *Acetoanaerobium* sp. which have been reported to utilize H_2_ and CO_2_ for acetate production^28^, although this community was not part of the core dominant community. It may also have arisen due to the presence of fermentative communities belonging to phyla Bacteroidetes, Synergistetes, Firmicutes, and Chloroflexi, which are known to degrade amino acids into acetate, H_2_ and CO_2_ from endogenous decay of the biofilm^23,45,46^. It is also possible that the consumption of the VFAs by the syntrophic members of the biocathodic community decreased in response to the change in potential, thereby allowing the VFAs to reach detectable limits. The mixotrophic *Methanosarcina* sp., which was present in the enriched biocathodes, can use acetate for acetoclastic methanogenesis and are capable of direct electron transfer due to their transmembrane cytochromes^47–49^.

Overall, the mixed nature of the biocathodic microbial community leads to difficulties in elucidating the production and consumption dynamics for *Methanobacterium* sp. strain 34x. Sulfate-reducing bacteria (SRB) of the genus *Desulfovibrio*, which have been shown to participate in direct electron transfer to produce H_2_ in the absence of sulfate in syntrophy with methanogens^50,51^, may have contributed to additional H_2_ production to support *Methanobacterium* sp. strain 34 × CH_4_ production independent of abiotic HER. The fermentative communities mentioned above produce H_2_ as a by-product of amino acid fermentation as well^23,47,48^. Since H_2_ is a ubiquitous electron donor, other microbial communities would have acted as electron sinks, thereby reducing the overall r_catCH4_ as well. The activity of methanogenic species other than *Methanobacterium* sp. strain 34x (Fig. 1 and Supplementary Table S2), while contributing to the overall CH_4_ production, was not captured in the metatranscriptional analysis due to limitations in the metagenomic resolution. Therefore, coupling 16S rRNA amplicon sequencing with metagenomic analysis allows for better identification of the microbial diversity in the enriched biocathodes as well as the transcriptional dynamics of the most functionally active and abundant species. As a PCR-based method, amplicon sequencing suffers from PCR bias, incomplete primer coverage and copy number differences between microorganisms^52^. However, amplicon sequencing is still useful due to extensive reference databases that allow for better identification of unknown microorganisms at lower abundance, although it is limited to genus-level identification. Additionally, functionality of unknown species can be inferred based on similarity to existing reference microorganisms^53^. Metagenomic analysis is useful for metabolic function analysis at a whole genome level^54^. However, in an enriched community, the lower abundance species are not fully captured. Presenting both 16S rRNA amplicon sequencing and metagenomic results provides a better view of the microbial communities present in the enriched biocathodes, which will allow us to better understand the possible product production/consumption dynamics.

### The metabolic pathways and gene expression

Methanogens conserve energy through CH_4_ production. In hydrogenotrophic methanogenesis, this is a 7-step biochemical pathway (Wolfe cycle) with a total standard free energy change (ΔGº’) of − 131 kJ/mol (Supplementary Table S8). These reactions require the input of 8 electrons for every CH_4_ molecule produced. H_2_ is oxidized by hydrogenases, which include the ferredoxin-reducing hydrogenases Eha and Ehc and the F_420_-reducing hydrogenase Frh (Reactions 8 and 9, Supplementary Table S8). ATP synthesis (Reaction 12) is coupled to this process via electrochemical Na + ion and proton gradients driven by methyl- H_4_MPT:coenzyme M methyltransferase (Mtr) (Reaction 6) and the electron-bifurcating hydrogenase:heterodisulfide reductase complex (Mvh:HdrABC, Reactions 9 and 10). The amount of ATP generated from hydrogenotrophic methanogenesis is 0.5 mol per 1 mol CH_4_^48^. All methanogenesis pathways involve a final step catalyzed by methyl-coenzyme reductase (Mcr) to reduce methyl-CoM to CH_4_, and the regeneration of coenzyme M (CoM) and coenzyme B (CoB).

Our reconstructed metabolic pathway (Fig. 4) is a summary of the observed differences in the expression of the genes involved in both methane generation and carbon metabolism in response to the changes in cathode set potential. The genes encoding enzymes involved in the early steps (Reactions 1–4, Supplementary Table S8) of the hydrogenotrophic methanogenesis pathway were at basal expression levels, with formyl-methanofuran dehydrogenase (*fwd*), F_420_-dependent methylene- H_4_MPT dehydrogenase (*mtd*) and F_420_-dependent methylene- H_4_MPT reductase (*mer*) present at higher basal expression compared to Formyl-methanofuran: H_4_MPT formyl transferase (*ftr*) and methenyl-H_4_MPT cyclohydrolase (*mch)*. The later steps were more highly expressed throughout the potential changes (Reactions 5, 7 and 9, Fig. 4 and Supplementary Table S8). Mcr (Reaction 7) and the Hdr complex (Reaction 9) were the most highly expressed within the methane metabolism pathway, with decreasing Mcr over time at -0.7 V compared to the baseline -1.0 V conditions. Since Mcr catalyzes the final step in CO_2_ reduction to CH_4_, lower Mcr would lead to lower CH_4_ production. That was indeed the case; by 90 min no CH_4_ was detected in the reactors (Table 1). Methanogenesis requires the presence of cofactors including coenzymes M, B and F_420_. The low and unchanged basal expression of the coenzyme pathways in response to the potential-induced changes could be because coenzymes M and F_420_ can be regenerated by H_2_, so they do not need to be continuously expressed to conserve energy^38^.

While the metatranscriptional analysis did not reveal any significant differential expression between the set cathode potentials tested, this does not necessarily mean that cathode potential does not affect gene expression in *Methanobacterium* sp. strain 34x. The lack of significant changes may have been due to the presence of H_2_-limited conditions even during the − 1.0 V operation. This is supported by the continuous expression of the Mcr isoenzyme MRI (*mcrABC*), which has a lower specific activity (K_m_ = 0.6–0.8 mM) and higher affinity (V_max_ = 6 µmol/min × mg^−1^) for H_2_ compared to Mcr isoenzyme MR II (K_m_ = 1.3–1.5 mM, V_max_ = 21 6 µmol/min × mg^−1^)^55^, which was not expressed in our system. While MRI and MRII are both expressed under non-H_2_ limiting conditions^56^, preferential expression of MRI under H_2_-limited conditions, due to its higher affinity for H_2_, was reported in *Methanothermobacter thermoautotrophicus,* another hydrogenotrophic methanogen. Due to its lower K_m_ for H_2_, high expression levels of MRI were detected in this study even in the absence of detectable CH_4_ after 90 min of − 0.7 V set potential-induced growth. It may be that *Methanobacterium* sp. strain 34 × MRI has a higher affinity for H_2_, allowing it to out-compete other methanogens under the observed H_2_-limited conditions. Further investigations are required to determine the enzymatic kinetics for this novel species to confirm if this is the reason for dominance in the enriched biocathodes.

### High relative expression of Acetyl-CoA synthetase (ACS)

Just as the availability of H_2_ somewhat affected the expression profile of *Methanobacterium* sp. strain 34x, the effect of acetate was also evident in the elevated expression of acetyl-CoA synthetase (ACS). Hydrogenotrophs can use acetate as a carbon source for growth in the presence of H_2_/CO_2_ where it is activated by ACS into acetyl-CoA and carboxylated to form pyruvate for carbon assimilation^57–62^. This is known as the reductive acetyl-CoA pathway^36,37,59,60,63–66^, where ACS activates one molecule of acetate or two molecules of CO_2_ into one molecule of acetyl-CoA using a coenzyme and enzyme metal center as the CO_2_ acceptors (Fig. 4). One of the CO_2_ molecules is reduced to a methyl group, which can then be bound to the H_4_MPT coenzyme. The second CO_2_ molecule is reduced to CO which is bound to Ni in the active site of CO dehydrogenase:acetyl-CoA synthase complex (ACDS). The methyl group in methyl-H_4_MPT combines with the bound CO in the CODH to release acetyl-CoA (Fig. 4 and Reaction 12 in Supplementary Table S8). Although this reductive acetyl-CoA pathway has a low energetic cost (~ 1 ATP molecule to synthesize 1 molecule of pyruvate), there are additional energetic requirements for the synthesis of the cofactors involved^63^. The resulting acetyl-CoA can then enter the biosynthetic pathway by being converted to pyruvate, an important intermediate in cell synthesis, by pyruvate synthase (POR)^57,63,67,68^ or it may be channeled into methanogenesis as methenyl-H_4_MPT (acetoclastic methanogenesis) (Reaction 4 in Supplementary Table S8), or CO for carboxydotrophic methanogenesis^22^. While both CO_2_ and acetate can be used for biosynthesis by ACS it is energetically more costly to produce pyruvate from acetate (7.69 kJ/e eq) compared to CO_2_ 114 kJ/e equivalent)^69^. This may have been the case for *Methanobacterium* sp. 34 × under the added energy constraints imposed due to further H_2_-limitation at -0.7 V. Interestingly, the highest expression observed overall for *Methanobacterium* sp. 34 × was for ACS, while POR and Cdh were also highly transcribed under all conditions (Fig. 4). Although not significantly differentially expressed, the expression of these genes was relatively higher at − 0.7 V after 90 min potential-induced growth compared to the baseline at − 1.0 V and − 0.7 V after only 45 min. n hydrogenotrophic methanogens, the acetyl-CoA reductive pathway has only been reported to contribute to carbon assimilation, although it is also linked to CH_4_ metabolism indirectly through formate. Coupled with the functional performance data, it is likely that the metabolic processes in *Methanobacterium* sp. strain 34 × were somewhat diverted more towards central carbon metabolism. This may have been due to the increased levels of acetate prompting a metabolic switch in response to the cathode potential changes^35^.

### Formate as an indirect electron donor

Acetate can be activated to acetyl-CoA, which can then be converted to pyruvate by POR and then formate by pyruvate formate lyase activating enzyme (PFL). Formate is oxidized by coenzyme F_420_-dependent formate dehydrogenase (Fdh, Eqyt. 4) and F420--reducing hydrogenase (Frh, Eq. 5) to CO_2_ and H_2_, thereby entering the CH_4_ pathway as an indirect electron donor^48,70^.

4 $$ {\text{HCOO}}^{-} + {\text{H}}^{ + } + {\text{F}}_{{{42}0}} \rightleftharpoons {\text{CO}}_{{2}} + {\text{ F}}_{{{42}0}} {\text{H}}_{{2}} \;\,\;\Delta G^{{ \circ^{\prime}}} = {-}{14 }\;{\text{kJ}}\;{\text{ per}}\;{\text{ mol}} $$

5 $$ {\text{F}}_{{{42}0}} {\text{H}}_{{2}} \rightleftharpoons {\text{F}}_{{{42}0}} + {\text{H}}_{{2}} \;\;\; \Delta G^{{ \circ^{\prime}}} = + {11}\;{\text{kJ}}\;{\text{per}}\;{\text{mole}} $$

The dynamics of formate concentration throughout MES operation and the potential-induced changes indicated both the production and consumption of formate by the biocathodic community. However, it is uncertain if formate was being used as an alternative electron donor under the H_2_-limited conditions imposed by -0.7 V specifically by *Methanobacterium* sp. strain 34x. Not all hydrogenotrophic methanogens can use formate as an electron donor, even though the *fdh* gene may be present^71^ or expressed^72^. The expression of *fdh* is H_2_-regulated, with maximal expression observed in the absence of H_2_^70^. Thus, it would have been expected to see an increase in *fdh* expression over the 45 min and 90 min operation at -0.7 V, whereas *fdh* was present at basal expression levels throughout the changes and no CH_4_ was detected after 90 min. Additionally, the absence of Frh, which is needed to regenerate the coenzyme F_420_, suggests that *Methanobacterium* sp. strain 34 × may not have been utilizing formate as an electron donor. It is possible that other members of the microbial consortia present at low relative abundance were involved in formate consumption, including other *Methanobacterium*^73^ and *Methanobrevibacter* sp.^74^. *Desulfovibrio* sp., which were present in low relative abundance on the biocathodes (Fig. 1), have also been shown to be capable of using formate to produce H_2_ in the absence of sulfate^75,76^. Unfortunately, these species could not be resolved into bins by the metagenomic analysis for further investigation.

### The reductive acetyl-coA pathway: acetoclastic methanogenesis or carbon assimilation?

It is unclear why, despite having the full complement of both hydrogenotrophic and acetoclastic methanogenesis pathways, hydrogenotrophic methanogens are not reported to utilize acetate as a source of energy for CH_4_ generation. This may also be due to the cyclic nature of hydrogenotrophic methanogenesis. The stoichiometric coupling of the Hdr complex with the first step of the pathway requires two pairs of electrons to regenerate CoB and CoM, as well as to reduce CO_2_ to formyl-MFR. However, acetate only provides one pair of electrons from its oxidation to carbonyl through the reductive acetyl-CoA pathway^77^. Richards et al.^38^ further investigated this by creating a metabolic reconstruction of the central energy-generating electron bifurcation reaction and carbon assimilation pathways in *M. maripaludis* as a model hydrogenotrophic methanogen*.* They demonstrated that the energy-converting hydrogenases (Eha and Ehb) required for the acetoclastic pathway could not function in a central stoichiometric role due to limitations on their overall electron flux. Eha/Ehb could only support the production of 10% CH_4_, supporting their anaplerotic role. In agreement, our results showed that *eha* expression was not high, and did not appear to respond to changes in set potential, as previously demonstrated^26^. This supports that the role of acetate and acetyl CoA was not towards methanogenesis but rather carbon assimilation as has been shown in other hydrogenotrophic methanogens. The high level of transcription for energy-consuming biosynthetic functions suggests the production of other proteins in response to the substrate limitation, which require further proteomic studies to fully understand. Limited isotopic studies using labeled acetate have been done to track the role of acetate in hydrogenotrophic methanogen biosynthetic processes^61,78^, including a report which determined that < 1% of labeled acetate was recovered as CH_4_^59^. Further isotopic studies coupled with transcriptional and proteomic analyses are needed with a focus on *Methanobacterium* sp. as dominant electromethanogenic communities^12,39^ to investigate what role acetate may play in hydrogenotrophic methanogenesis.

Despite the obvious differences in current density and the associated H_2_ and CH_4_ production, we did not observe significant differential gene expression. These results are in line with the findings from a recent report by Perona-Vico et al.^26^ which found no statistically significant differential gene expression in a *Methanobacterium* sp.*-*dominant biocathode that was operated under − 1.0 V (vs. Ag/AgCl) and open-circuit voltage for 6 h^26^. However, clearly the methanogenesis pathway was affected in our system, since after 90 min of potential-induced growth at − 0.7 V, no CH_4_ was detected in any of the replicates (Table 1). This suggests that either *Methanobacterium* sp. strain 34 × was not directly affected at a transcriptional level by H_2_ availability, or that the changes in H_2_ availability were not large enough to elicit a significant response from the basal expression, or that broader sampling and testing conditions were required to observe differential expression, or a combination of these possibilities. The high number of genes expressed involved in translation (90 genes) compared to transcription (18 genes) supports the need for proteomic and isotopic studies to fully understand the functional response of electromethanogenic communities in response to set potential changes.

## Materials and methods

### MES set up and operation

Triplicate double-chambered MES reactors were prepared using 300 ml screw-capped borosilicate glass bottles with a working volume of 280 ml, separated by a Nafion 117 cation exchange membrane (5 cm^2^, Sigma, USA)^79^. The caps and bottles were modified with appropriate ports to place the electrodes, gas collection bags (Calibrate, Inc., USA) and gas sampling ports. The ports were 2 cm long, with a diameter of 0.5 cm. The anodes were abiotic titanium plates using titanium wires as the current collectors^79^. The cathodes were carbon cloth (8 cm length × 10 cm width, 160 cm^2^ geometric surface area), previously enriched in microbial electrolysis cells using sludge (10% v/v) from an anaerobic membrane bioreactor treating synthetic municipal wastewater as an initial inoculum. Teflon tape and epoxy were applied on all the connections to ensure a proper seal. The reactors were batch-fed with each batch lasting 140 h. At the end of each batch, the media were replaced with influent media prepared using a modified DSMZ Medium 826 protocol (DSMZ, Leibniz, Germany) with the following composition (g/L): NH_4_Cl, 1.5; Na_2_HPO_4_, 0.6; KCl, 0.1; Na_2_HCO_3_, and 10 ml trace minerals and vitamin solution each^79^. To maintain anaerobic conditions, the media was sparged for 1 h using a N_2_:CO_2_ (80:20) gas mixture and then autoclaved. Sodium bicarbonate was sterile filtered into the media after autoclaving to maintain a pH of ~ 7.5. The only carbon source was CO_2_ in the form of dissolved sodium bicarbonate for pH adjustment and 100% CO_2_ gas, which was continuously bubbled into the reactors at the beginning of each batch for 5 min and acted as a CO_2_ reservoir through passive gas diffusion from the gasbags into the reactor headspace. An Ag/AgCl reference electrode (BASi, USA) was inserted in the cathode chamber to maintain the set potential control. A VMP3 potentiostat (BioLogic, USA) was connected to the three-electrode system to chronoamperometrically maintain a cathode set potential of − 1.0 V vs. Ag/AgCl.

### Set potential changes and sampling

All reactors were maintained at the same set cathode potentials to allow for triplicate samples which are required for metatranscriptomics analyses to determine statistical significance. Once stable methane production was observed for three consecutive batches, the set potential changes and sampling were started. Before the start of the set potential experiment, baseline biofilm samples were taken from all the biocathodes for protein, ATP and all microbial community analyses. This was followed by replacing the cathode media and continuously flushing the headspace for 5 min with 100% CO_2_. The reactors were then connected to the potentiostat and maintained at − 1.0 V for 1 h to allow for current density recovery and CH_4_ detection from the baseline sampling event, as determined by previous time course experiments (data not shown). The reactor headspaces were once again flushed with 100% CO_2_ and the set cathode potential was switched to − 0.7 V. After 45 min, the second sampling took place to represent the − 0.7 V sample point. After the second sampling event, the cathode media was replaced, the headspace flushed with 100% CO_2_ and the reactors were reconnected to the potentiostat and maintained at − 0.7 V for another 45 min after which the final sampling event took place. 45 min was chosen as the first sampling point after the cathode set potential change based on previous studies investigating microbial response to potential changes in METs^80,81^. Further, CH_4_ was detectable in the systems within 45 min of batch change in our preliminary time series trials (data not shown). CH_4_ production was considered an indicator of the CH4-related expression profile. Additionally, mRNA are known to have short half-lives ranging in minutes^82,83^. All biocathode samples were collected from three random points by cutting using sterilized scissors, with a total of ~ 6 cm^2^ taken from each reactor for every sampling event. The triplicate samples were pooled in a tube into one sample for each reactor containing 6 ml sterile media with an equal amount of RNAlater to prevent RNA degradation and immediately placed in liquid nitrogen for 1 min to arrest any further RNA transcription. These samples were subjected to vortexing for 1 min to detach the microbial cells from the cathode and stored at − 20 °C for subsequent DNA/RNA co-extraction for amplicon sequencing and metagenomics and metatranscriptomics analyses. A sample from the anaerobic sludge was stored at -20 °C for amplicon and metagenomic analyses as well.

### Measurement and analyses

Liquid and gas (H_2_, CH_4_ and CO_2_) samples were measured at the end of each batch cycle using chromatographic methods. Samples were filtered through 0.2 µm filters prior to analysis. Volatile fatty acids were detected at 210 nm using an Aminex HP-87H column (Bio-Rad, Hercules, CA) with a UV-detector high performance liquid chromatography (HPLC; Shimadzu, Japan). The mobile phase was 0.005 M H_2_SO_4_ at a flow rate of 0.55 ml/min. The gas compositions in the headspaces and gas bags were analyzed using a gas chromatograph (GC; Model# 8610C, SRI Instruments, USA) as previously described^84^. During the set potential sampling experiment, gas and liquid samples were taken at the start and end of each operational change.

### MES calculations

Current density was calculated as the recorded current (mA) divided by the geometric surface area of the cathode (160 cm^2^). Cathodic conversion efficiencies in terms of hydrogen, methane, formate and acetate recovery (r_catH2_, r_catCH4,_ r_catform_, r_catace_) were calculated by:

$$ {\text{r}}_{{{\text{cat}}}} { = }\frac{{\text{n}}}{{{\text{n}}_{{{\text{CCE}}}} }} $$

where n is the measured moles of the specific product (H_2_, CH_4_, formate or acetate). n_CCE_ is the total moles of specific product possible based on the total coulombs C_t_ as recorded by the VMP3 software, and is calculated by:

$$ {\text{n}}_{{{\text{CCE}}}} { = }\frac{{{\text{C}}_{{\text{t}}} }}{{{\text{bF}}}} $$

where b is the number of moles of electrons required for hydrogen production (2 mol e^−^), methane production (8 mol e^−^), formate production (2 mol e^−^), and acetate production (8 mol e^−^), and F is Faraday’s constant (96,485 C/mol e^−^).

### Microbial community analyses

#### DNA/RNA co-extraction

Genomic DNA was co-extracted with RNA using the PowerBiofilm RNA Isolation Kit (Qiagen, Germany) with a modified protocol using phenol:chloroform:isoamyl alcohol pH 6.5–8.0 (AMRESCO, Inc., USA) and bead beating lysing matrix E tubes (MP Biomedicals, New Zealand). The extracted DNA concentration was measured using Qubit dsDNA HS Assay Kit (Thermo Scientific, USA) and the RNA concentration was measured using the Qubit RNA HS Assay Kit (Thermo Scientific, USA), according to the manufacturer’s instructions.

#### 16S rRNA gene library preparation, sequencing and bioinformatics processing

Amplicon libraries were prepared for the archaeal and bacterial 16S rRNA gene V3-V4 region using up to 10 ng of the extracted DNA, the forward primer Pro341F (5′-CCTACGGGNBGCASCAG-3′) and the reverse primer Pro805R (5′-GACTACNVGGGTATCTAATCC-3′)^85^. Each PCR reaction (25 μL) contained dNTPs (100 μM of each), MgSO_4_ (1.5 mM), Platinum Taq DNA polymerase HF (0.5 U/reaction), Platinum High Fidelity buffer (1X) (Thermo Fisher Scientific, USA) and tailed primer mix (400 nM of each forward and reverse primer). The PCR amplification was conducted by an initial denaturation step at 95 °C for 2 min, 35 cycles of amplification (95 °C for 20 s, 50 °C for 30 s, 72 °C for 60 s) and a final elongation at 72 °C for 5 min^86^. Duplicate PCR reactions were performed for each sample and the duplicates were pooled after PCR. The resulting amplicon libraries were purified using the standard protocol for Agencourt Ampure XP Beads (Beckman Coulter, USA) with a bead to sample ratio of 4:5. DNA concentrations were measured using the Qubit dsDNA HS Assay Kit, followed by product size and purity validation with gel electrophoresis using Tapestation 2200 and D1000/High sensitivity D1000 screentapes (Agilent, USA).

Sequencing libraries were prepared from the purified amplicon libraries using a second PCR. Each PCR reaction (25 μL) contained PCRBIO HiFi buffer (1x), PCRBIO HiFi Polymerase (1 U/reaction) (PCRBiosystems, UK), adaptor mix (400 nM of each forward and reverse) and up to 10 ng of amplicon library template. The PCR amplification was conducted by an initial denaturation step at 95 °C for 2 min, 8 cycles of amplification (95 °C for 20 s, 55 °C for 30 s, 72 °C for 60 s) and a final elongation at 72 °C for 5 min. The resulting sequencing libraries were purified as mentioned above using the Agencourt Ampure XP Beads. DNA concentration, product size and purity were measured as mentioned above. The purified sequencing libraries were pooled in equimolar concentrations and diluted to 2 nM. The samples were paired-end sequenced (2 × 300 bp) on a MiSeq using a MiSeq Reagent kit v3 (Illumina, USA) following the standard guidelines for preparing and loading samples on the MiSeq. > 10% PhiX control library was spiked in to overcome low complexity issues often observed with amplicon samples.

Forward and reverse reads were trimmed for quality using Trimmomatic v. 0.32^87^ with the settings SLIDINGWINDOW:5:3 and MINLEN: 275. The trimmed forward and reverse reads were merged using FLASH v. 1.2.7^88^ with the settings -m 10 -M 250. The trimmed reads were dereplicated and formatted for use in the UPARSE workflow^89^. The dereplicated reads were clustered, using the usearch v. 7.0.1090 -cluster_otus command with default settings. Operational taxonomic unit (OTU) abundances were estimated using the usearch v. 7.0.1090 -usearch_global command with -id 0.97 -maxaccepts 0 -maxrejects 0. Taxonomy was assigned using the RDP classifier^90^ as implemented in the parallel_assign_taxonomy_rdp.py script in QIIME^91^, using –confidence 0.8 and the MiDAS database (v. 1.23)^92^, which is a curated database based on the SILVA database (release 123)^93^. The results were analyzed in R v. 3.5.0^94^.

#### Metagenome library preparation, sequencing and bioinformatic processing

The DNA was fragmented to approximately 550 bp using a Covaris M220 with microTUBE AFA Fiber screw tubes at 200 cycles/burst for 45 s with a Duty Factor of 20% and Peak/Displayed Power of 50 W. The fragmented DNA was used for metagenome preparation using the NEB Next Ultra II DNA library preparation kit (New England BioLabs, USA). The DNA library was paired-end sequenced (2 × 150 bp) on a NextSeq system (Illumina, USA).

The sequence reads were trimmed for adaptors using cutadapt (v. 1.10)^95^ and assembled using SPAdes (v. 3.7.1)^96^. The reads were mapped back to the assembly using minimap2 (v. 2.5)^97^ to generate coverage files for metagenomic binning. The 16S rRNA genes were identified using BLASTn (v. 2.2.28 +)^98^, and the 16S rRNA fragments were classified using SINA (v. 1.2.11)^99^with the min identity adjusted to 0.80 but otherwise default settings. The supporting data for binning was generated according to the description in the mmgenome package (v. 0.7.1)^100^. Genome binning was carried out in R (v. 3.3.4)^94^, refined manually as described in the mmgenome package and the final bins were annotated using Prokka (v1.12)^101^.

Average Nucleotide Identity (ANI) between the extracted bin and the genomes of representative species of *Methanobacterium* was calculated with OrthoANI^102^. The cut-off threshold for species delineation was ≥ 97%^103^. For phylogenomic analyses, available genomes assemblies of *Methanobacterium* were downloaded from the NCBI GenBank (June, 2019). Hidden Markov model profiles for 139 single-copy core genes^104^ were concatenated using the anvi’o platform^105^. Phylogenetic trees with estimated branch support values were constructed from these concatenated alignments using MEGA7^32^ with Neighbor-Joining and Maximum-likelihood. The evolutionary distances were computed using the Poisson correction method^31^ and are in the units of the number of amino acid substitutions per site. All positions with less than 95% site coverage were eliminated. That is, fewer than 5% alignment gaps, missing data, and ambiguous bases were allowed at any position.

#### Metatranscriptome library preparation, sequencing and bioinformatic processing

RNA quality was confirmed using TapeStation with RNA ScreenTape (Agilent Technologies). The samples were depleted from rRNA using the Ribo-zero Magnetic kit (Illumina, USA) according to the manufacturer’s instructions. Any potential residual DNA was removed using the DNase MAX kit (MoBio Laboratories Inc., Germany). After rRNA depletion and DNase treatment, the samples were cleaned and concentrated using the RNeasy MinElute Cleanup kit (QIAGEN, Germany) and successful rRNA removal was confirmed using TapeStation HS RNA Screentapes (Agilent Technologies, USA). The samples were prepared for sequencing using the TruSeq Stranded Total RNA kit (Illumina, USA) according to the manufacturer’s instructions. Library concentrations were measured using Qubit HS DNA assay and library size estimated using TapeStation D1000 ScreenTapes (Agilent Technologies, USA). The samples were sequenced on an Illumina HiSeq2500 using a 1 × 50 bp Rapid Run (Illumina, USA).

Raw sequence reads in fastq format were trimmed using USEARCH (v10.0.2132)^106^-fastq_filter with the settings -fastq_minlen 45 -fastq_truncqual 20. The trimmed transcriptome reads were depleted of rRNA using BBDuk^107^ using the SILVA database as reference database^93^. The reads were then mapped to the predicted protein coding genes generated from Prokka (v1.12)^101^ using minimap2 (v2.8-r672)^97^, both for the total metagenome and each genome bin. Reads with a sequence identity below 0.98 were discarded. To identify differentially expressed genes, the count tables were imported to R^94^, processed using the DESeq2 workflow^108^ and visualized using ggplot2. Transcriptional counts were normalized to transcripts per million (TPM). TPM was calculated by dividing the read counts by the length of each gene in kilobases to get reads per kilobases (RPK). All the RPK values were added for each sample and divided by 1 million to get the “per million” scaling factor. Finally, the RPK values were divided by the “per million” scaling factor to yield the TPM values^109^. To reconstruct the metabolic pathways present in the extracted genome, Kegg Orthology (KO) identifiers/homologs were assigned to the genome with BlastKOALA^110^. Subcellular location of the proteins was predicted with PsortB (v3.0)^111^. Pathway expression was calculated as the average expression of the steps within a pathway. If a pathway step included an enzyme complex, the average expression of each subunit was used as the expression value for that step. If a reaction could be catalyzed by more than one enzyme, or if multiple copies of an enzyme were encoded by the genome, the summed expression of the enzymes or copies was used as the expression value for that step^112^.

### Statistical analyses

Statistical analyses were performed in RStudio IDE using the base R^113^. The normality of data distribution was examined by the Shapiro–Wilk test. One-way analysis of variance (ANOVA) was used to compare parametric variables among three or more groups, and the Kruskal–Wallis test was used for nonparametric variables. The two-tailed (independent) Student’s *t*-test was used to compare means between unpaired groups with an assumption of unequal variance between sample sets. The Mann–Whitney *U*-test was used to compare nonparametric variables between two groups. Quantitative variables were expressed as the mean ± standard deviation. *P*-values less than 0.05 were considered to indicate statistical significance against the null hypothesis of no variance.

## Supplementary information

Supplementary Information 1.

Supplementary Information 2.

## Data Availability

The 16S rRNA gene sequencing reads are deposited in the National Center for Biotechnology Information (NCBI) under BioProject ID PRJNA543631 with accession numbers SAMN12169213—SAMN12169222. The GenBank file of the extracted *Methanobacterium* sp. strain 34 × draft genome from this study is filed under the accession number VCMF00000000.1. The Sequence Read Archive (SRA) accession numbers are SRR9192478 and SRR9192479. The genome binning and differential expression analyses are entirely reproducible using the R files available at https://github.com/DarioRShaw/Cathode-set-potential.
