# Supplementary material for: Effects of set cathode potentials on microbial electrosynthesis system performance and biocathode methanogen function at a metatranscriptional level
Source: Sci Rep. 2020 Nov 13;10:19824. doi: 10.1038/s41598-020-76229-5 (PMC7666199; doi:10.1038/s41598-020-76229-5)
Supplement: Supplementary file 1 — Supplementary Information 1. [file 41598_2020_76229_MOESM1_ESM.pdf]

Supplementary Information

Effects of set cathode potentials on microbial electrosynthesis system performance and biocathode methanogen function at a metatranscriptional level

Ala'a Ragab[1], Dario Rangel Shaw[1], Krishna Katuri[1], Pascal E. Saikaly[1,*]

[1]Biological and Environmental Science and Engineering Division, Water Desalination and Reuse Center, King Abdullah University of Science and Technology (KAUST), Saudi Arabia

*** Correspondence:** Pascal E. Saikaly; pascal.saikaly@kaust.edu.sa

Table S1 | Alpha diversity results for the three –1.0 V baseline samples, rarefied to 47,369 reads.

Reactor	Observed OTUs	Shannon	Simpson	Chao1
R1	129	2.52	6.99	132.24
R2	127	2.87	11.93	172.33
R3	103	2.24	4.22	105.00

Table S2 | 16S rRNA Amplicon sequencing operational taxonomic unit (OTU) relative abundances for the three –1.0 V baseline samples, with taxonomic classification to the lowest available level. The grey shaded cells are the core dominant community OTUs (≥ 0.1% in all samples) (See Supplementary Excel File).

Table S3 | Basic statistics of the extracted genome Bin 1. Number of scaffolds is the number of scaffolds in the extracted genome bin. N50 (bp) is a median statistic that indicates that 50% of the entire assembly is contained in scaffolds equal to or larger than this value (bp). Length Total (bp) is the total combined length of the extracted genome. Length max (bp) is the length of the largest scaffold in the extracted bin. Length mean is the mean length of the scaffolds in the extracted bin. Mean GC content (%) is the mean GC content of all scaffolds in the bin weighted by scaffold length. The cov_prefix shows the average coverage of the bin in all available samples. Total essential genes (#) is the total number of identified essential "single copy" genes in the genome bin. Unique essential genes (#) is the number of unique essential "single copy" genes.

General statistics	Value
Number of scaffolds	80
N50 (bp)	72243
Length Total (bp)	2245896
Length max (bp)	230406
Length mean (bp)	28073.7
Mean GC content (%)	36.95
cov_Baseline −1V_1 (% of reads mapped to Bin 1)	83.22
cov_Baseline −1V_2 (% of reads mapped to Bin 1)	68.43
cov_Baseline −1V_3 (% of reads mapped to Bin 1)	72.65
cov_Inoculum_0 (% of reads mapped to Bin 1)	0.61
Total essential genes (#)	35
Unique essential genes (#)	35

Table S4 | Average Nucleotide Identity by Orthology (OrthoANI) of the extracted Bin 1 compared to representative whole genome species.

Methanobacterium	**OrthoANI value (%)**
LT607756.1 *Methanobacterium congolense*	69.29
CP022706.1 *Methanobacterium* sp. BAmetb5	69.69
CP022705.1 *Methanobacterium* sp. BRmetb2	69.08
CP017768.1 *Methanobacterium subterraneum* strain A8p	69.61
CP017767.1 *Methanobacterium* sp. MZ-A1	69.66
CP017766.1 *Methanobacterium* sp. MO-MB1	69.72
LN515531.1 *Methanobacterium formicicum* genome assembly DSM1535	69.03
CP006933.1 *Methanobacterium formicicum* strain BRM9	69.21
HG425166.1 *Methanobacterium* sp. MB1	69.45
CP002772.1 *Methanobacterium paludis* strain SWAN1	69.68
CP002551.1 *Methanobacterium lacus* strain AL-21	68.98

Table S5 | Full list of functionally annotated genes. (See Supplementary Excel file)

Table S6 | Bin 1 metatranscriptomic sequencing statistics.

	**–1.0 V, baseline**			**–0.7 V, 45 min**			**–0.7 V, 90 min**		
	**R1**	**R2**	**R3**	**R1**	**R2**	**R3**	**R1**	**R2**	**R3**
**Total raw reads**	23882232	23007256	17605921	25032345	24847101	20780365	16870228	20775674	21255588
**Reads after filtering**	23473030	22525809	17246244	24270527	24446390	20456427	16552288	20381947	20908929
**Total mapped reads**	19285676	18076847	13060741	20553437	17155122	15853567	12783523	16081855	16563685
**Non-rRNA mapped reads**	8082943	8666373	7847438	6542286	11013536	10811475	7127615	8607688	11731518
**Reads mapped to Bin 1**	7249249	7429946	6087028	6196525	8829602	9732602	6425014	7688412	11249856
**% non-rRNA reads mapped to Bin 1**	89.69	85.73	77.57	94.71	80.17	90.02	90.14	89.32	95.89

Table S7 | Expression counts in transcripts per million (TPM) for the proteins shown in the reconstructed metabolic pathway for the three different sample points (–1.0 V baseline, –0.7 V for 45 min, –0.7 V for 90 min) for the three reactors. The proteins shaded in orange belong to hydrogenotrophic methanogenesis methane metabolism pathway, the green shading denotes proteins that are part of carbon fixation and the unshaded area denotes proteins involved in co-enzyme metabolism associated with methanogenesis.

			-1.0 V, baseline			-0.7 V, 45 min			-0.7 V, 90 min		
**Abbreviation in graphical model**	**Protein**	**locus tag**	**R1 (TPM)**	**R2 (TPM)**	**R3 (TPM)**	**R1 (TPM)**	**R2 (TPM)**	**R3 (TPM)**	**R1 (TPM)**	**R2 (TPM)**	**R3 (TPM)**
**Fwd**	fwdA, fmdA; formylmethanofuran dehydrogenase subunit A [EC:1.2.7.12]	FGO69_07490	912.9	460.4	695.0	1137.9	500.0	604.0	1607.9	671.5	631.0
		FGO69_09480	1258.8	594.0	1615.0	555.5	715.1	613.6	1166.3	642.2	852.3
	fwdB, fmdB; formylmethanofuran dehydrogenase subunit B [EC:1.2.7.12]	FGO69_07485	611.6	329.8	475.1	781.2	350.7	398.5	1036.5	485.3	431.3
		FGO69_09475	828.8	369.6	863.9	416.1	436.0	397.2	738.3	388.7	535.5
	fwdC, fmdC; formylmethanofuran dehydrogenase subunit C [EC:1.2.7.12]	FGO69_01970	311.9	315.0	397.7	401.7	349.1	365.0	325.6	363.8	380.0
		FGO69_07495	1153.0	617.9	892.9	1016.3	639.2	560.8	1684.0	776.0	582.9
		FGO69_09485	1153.7	619.3	1093.0	741.0	681.9	546.2	1420.4	723.6	640.1
	fwdD, fmdD; formylmethanofuran dehydrogenase subunit D [EC:1.2.7.12]	FGO69_07480	716.4	348.1	407.5	826.1	379.3	337.9	1054.1	473.6	353.6
		FGO69_09470	902.8	414.3	740.2	475.6	477.8	349.0	711.1	405.6	467.8
	fwdE, fmdE; formylmethanofuran dehydrogenase subunit E [EC:1.2.7.12]	FGO69_01135	361.4	344.3	210.7	233.4	275.2	308.5	294.5	216.8	277.8
		FGO69_07550	55.9	12.2	121.5	14.9	11.7	17.9	10.7	12.5	17.9
		FGO69_07530	90.5	74.5	116.5	81.2	71.8	76.0	77.0	81.8	74.4
		FGO69_09520	70.0	53.3	76.9	23.8	50.6	41.0	32.8	41.2	36.0
		FGO69_10815	12.5	11.8	26.0	12.1	14.4	16.4	6.9	12.7	13.2
		FGO69_11545	22.6	11.2	70.6	7.3	12.5	6.1	6.1	8.0	10.9

	fwdF, fmdF; 4Fe-4S ferredoxin	FGO69_07470	1130.9	593.0	583.3	1557.6	750.1	562.1	2445.5	884.5	514.1
		FGO69_07570	318.9	715.2	611.0	360.5	685.3	912.9	405.5	898.5	786.3
		FGO69_09460	1026.6	465.3	727.1	593.4	615.9	399.3	1185.8	482.3	433.1
**Ftr**	ftr; formylmethanofuran--tetrahydromethanopterin N-formyltransferase [EC:2.3.1.101]	FGO69_02880	362.6	317.7	465.9	240.2	312.2	344.0	292.7	305.0	356.2
		FGO69_03710	106.8	53.2	259.2	38.0	55.9	80.3	40.9	62.8	110.6
**Mch**	mch; methenyltetrahydromethanopterin cyclohydrolase [EC:3.5.4.27]	FGO69_04225	312.4	334.5	375.0	233.0	336.3	310.1	340.6	331.0	306.3
**Mtd**	mtd; methylenetetrahydromethanopterin dehydrogenase [EC:1.5.98.1]	FGO69_08320	693.4	1010.1	2722.0	732.0	1297.9	2259.1	1203.2	1099.3	2271.4
**Mer**	mer; 5,10-methylenetetrahydromethanopterin reductase [EC:1.5.98.2]	FGO69_00185	1799.0	1128.2	5819.6	1233.3	1148.2	3438.1	2323.6	981.1	3526.0
**Mtr**	mtrA; tetrahydromethanopterin S-methyltransferase subunit A [EC:2.1.1.86]	FGO69_01045	1560.8	545.5	3030.5	1126.6	661.9	1165.3	2384.5	582.1	1317.2
	mtrB; tetrahydromethanopterin S-methyltransferase subunit B [EC:2.1.1.86]	FGO69_01050	913.5	244.4	1629.0	544.1	314.4	598.0	949.3	274.7	788.8
	mtrC; tetrahydromethanopterin S-methyltransferase subunit C [EC:2.1.1.86]	FGO69_01055	820.5	260.6	1732.4	504.9	333.7	657.6	863.1	318.5	910.4
	mtrD; tetrahydromethanopterin S-methyltransferase subunit D [EC:2.1.1.86]	FGO69_01060	769.4	337.9	1826.3	643.5	377.9	818.0	1059.5	313.8	859.9
	mtrE; tetrahydromethanopterin S-methyltransferase subunit E [EC:2.1.1.86]	FGO69_01065	881.1	293.7	1536.6	601.3	303.1	798.8	953.8	262.5	908.8
	mtrF; tetrahydromethanopterin S-methyltransferase subunit F [EC:2.1.1.86]	FGO69_01040	1984.2	568.4	2861.1	1350.8	682.4	1162.3	2840.5	646.6	1426.7

	mtrG; tetrahydromethanopterin S-methyltransferase subunit G [EC:2.1.1.86]	FGO69_01035	1582.5	469.9	1952.9	1063.7	612.6	1003.6	2216.9	510.5	1154.9
	mtrH; tetrahydromethanopterin S-methyltransferase subunit H [EC:2.1.1.86]	FGO69_01030	3447.0	1270.3	5464.3	2185.7	1508.6	2399.6	5019.5	1261.8	2465.4
**Mcr**	mcrA; methyl-coenzyme M reductase alpha subunit [EC:2.8.4.1]	FGO69_01070	6400.1	3143.7	24772.2	4652.5	3432.2	9032.0	9520.7	3041.4	10220.5
	mcrB; methyl-coenzyme M reductase beta subunit [EC:2.8.4.1]	FGO69_01090	4899.1	1522.7	12929.0	3147.1	1749.6	4876.7	5281.4	1541.9	6515.2
	mcrG; methyl-coenzyme M reductase gamma subunit [EC:2.8.4.1]	FGO69_01075	4248.8	1412.3	11362.3	2946.7	1618.5	4571.0	5639.8	1489.1	5836.7
**Hdr**	hdrA2; heterodisulfide reductase subunit A2 [EC:1.8.7.3, 1.8.98.4, 1.8.98.5, 1.8.98.6]	FGO69_03370	5669.6	7070.5	4750.9	5207.0	6744.7	6924.0	6909.9	8258.0	7591.8
		FGO69_03625	474.6	661.1	378.4	405.6	724.0	528.8	430.1	1021.1	472.7
	hdrB2; heterodisulfide reductase subunit B2 [EC:1.8.7.3, 1.8.98.4, 1.8.98.5, 1.8.98.6]	FGO69_01495	2051.1	952.2	2651.8	1779.4	1072.1	975.3	2787.9	1053.8	888.6
		FGO69_03630	379.0	433.4	270.4	385.2	522.9	285.7	316.5	718.8	238.5
	hdrC2; heterodisulfide reductase subunit C2 [EC:1.8.7.3, 1.8.98.4, 1.8.98.5, 1.8.98.6]	FGO69_01500	999.6	303.7	980.0	1680.0	708.6	352.6	1849.0	735.1	386.3
		FGO69_03635	406.9	486.7	185.5	488.2	547.3	328.8	289.7	695.7	262.6
**Mvh**	mvhA, vhuA, vhcA; F420-non-reducing hydrogenase large subunit [EC:1.12.99.- 1.8.98.5]	FGO69_00980	2047.5	1209.3	4416.1	1414.3	1413.7	3654.2	2760.3	1553.4	4249.4
		FGO69_01940	606.1	774.6	843.5	431.5	812.1	1043.3	666.0	842.2	1044.1
	mvhG, vhuG, vhcG; F420-non-reducing hydrogenase small subunit [EC:1.12.99.- 1.8.98.5]	FGO69_00985	1962.2	1109.2	4002.8	1584.8	1316.0	2608.9	2637.6	1481.4	3009.0
		FGO69_01935	611.1	781.4	824.6	500.7	868.0	1091.4	758.2	980.2	1060.3
	mvhD, vhuD, vhcD; F420-non-reducing hydrogenase iron-sulfur subunit [EC:1.12.99.- 1.8.98.5 1.8.98.6]	FGO69_00990	1306.8	745.6	2556.6	1166.3	908.7	1603.1	1678.6	902.3	1714.0

**ATPase**	ATPVI; V/A-type H+/Na+-transporting ATPase subunit I	FGO69_00650	147.7	92.4	310.1	105.1	113.0	172.9	152.9	87.9	146.2
	ATPVK; V/A-type H+/Na+-transporting ATPase subunit K	FGO69_00655	189.5	118.2	563.0	146.6	136.9	235.6	177.0	124.3	265.5
	ATPVE; V/A-type H+/Na+-transporting ATPase subunit E	FGO69_00660	148.7	59.6	290.1	70.7	71.9	128.2	103.0	71.4	133.9
	ATPVC; V/A-type H+/Na+-transporting ATPase subunit C	FGO69_00665	130.3	60.8	265.6	76.6	84.1	131.6	94.7	62.6	130.1
	ATPVF; V/A-type H+/Na+-transporting ATPase subunit F	FGO69_00670	161.9	69.6	352.7	80.1	84.8	113.2	93.3	54.4	127.8
	ATPVA; V/A-type H+/Na+-transporting ATPase subunit A [EC:7.1.2.2, 7.2.2.1]	FGO69_00675	142.2	82.4	509.1	89.1	100.1	169.7	122.6	100.4	207.9
	ATPVB; V/A-type H+/Na+-transporting ATPase subunit B	FGO69_00680	191.2	136.7	614.8	119.8	148.0	269.0	174.7	138.1	295.6
	ATPVD; V/A-type H+/Na+-transporting ATPase subunit D	FGO69_00685	130.7	68.3	412.9	53.1	62.8	138.0	81.4	58.7	136.0
**Eha**	ehaO; energy-converting hydrogenase A subunit O	FGO69_03730	482.4	381.6	567.2	340.4	427.7	452.9	475.4	439.4	471.0
	ehaL; energy-converting hydrogenase A subunit L	FGO69_03745	81.6	51.1	95.6	36.4	54.5	91.0	54.6	31.9	64.3
	ehaJ; energy-converting hydrogenase A subunit J	FGO69_03755	142.7	111.3	260.7	80.4	114.1	171.5	145.9	81.7	129.6
	ehaH; energy-converting hydrogenase A subunit H	FGO69_03765	198.0	117.9	344.0	132.7	126.4	204.0	168.2	117.6	172.0
	ehaG; energy-converting hydrogenase A subunit G	FGO69_03770	335.9	258.7	510.3	229.4	263.4	449.1	332.7	203.3	359.6
	ehaF; energy-converting hydrogenase A subunit F	FGO69_03775	503.1	483.8	659.8	438.0	554.9	749.9	566.2	403.2	632.7
	ehaE; energy-converting hydrogenase A subunit E	FGO69_03780	511.8	632.3	478.8	427.9	674.0	853.6	656.7	481.0	619.9
	ehaD; energy-converting hydrogenase A subunit D	FGO69_03785	467.0	645.3	331.5	371.0	630.4	709.7	586.3	467.8	552.0

	ehaC; energy-converting hydrogenase A subunit C	FGO69_03790	664.0	870.2	304.1	541.4	669.8	946.5	671.0	452.2	682.2
	ehaB; energy-converting hydrogenase A subunit B	FGO69_03795	1445.9	2270.0	1784.9	1187.0	1820.7	2541.9	1781.9	1677.6	2122.0
	ehaM; energy-converting hydrogenase A subunit M	FGO69_03740	300.4	149.8	385.2	131.5	144.2	206.1	173.5	108.0	151.0
**ACS**	ACS, acs; acetyl-CoA synthetase [EC:6.2.1.1]	FGO69_05315	12079.1	10766.2	12862.4	10482.9	10629.9	11954.2	12209.6	12030.0	13349.2
		FGO69_09990	325.3	543.4	395.6	230.6	519.9	684.0	326.8	572.7	646.0
**ACDS (acetyl-CoA decarbonylase/synthase (ACDS) complex)**	cdhC; acetyl-CoA decarbonylase/synthase, CODH/ACS complex subunit beta [EC:2.3.1.169]	FGO69_00320	424.2	819.8	742.2	344.9	963.2	1184.2	641.2	1204.6	1522.9
	cdhE, acsC; acetyl-CoA decarbonylase/synthase, CODH/ACS complex subunit gamma [EC:2.1.1.245]	FGO69_00335	563.1	1296.6	1237.1	438.2	1487.5	1971.3	800.6	2039.0	2679.3
	cdhD, acsD; acetyl-CoA decarbonylase/synthase, CODH/ACS complex subunit delta [EC:2.1.1.245]	FGO69_00330	504.0	1110.6	1079.1	410.7	1321.1	1611.9	803.3	1845.9	2267.1
**Cdh**	cdhA; anaerobic carbon-monoxide dehydrogenase, CODH/ACS complex subunit alpha [EC:1.2.7.4]	FGO69_00310	522.3	1222.8	1077.3	442.3	1366.4	2016.6	786.2	1728.3	2554.6
	cdhB; anaerobic carbon-monoxide dehydrogenase, CODH/ACS complex subunit epsilon	FGO69_00315	536.6	1059.5	1122.5	396.3	1160.7	1634.0	767.0	1635.5	2201.0
	cooF; anaerobic carbon-monoxide dehydrogenase iron sulfur subunit	FGO69_00300	1803.7	1961.6	1717.7	1499.1	2163.9	2524.8	2165.3	2797.4	3009.4
**POR**	porA; pyruvate ferredoxin oxidoreductase alpha subunit [EC:1.2.7.1]	FGO69_00290	4839.3	6805.9	5568.1	5170.7	7911.8	9065.3	6675.1	9900.8	11565.5
	porB; pyruvate ferredoxin oxidoreductase beta subunit [EC:1.2.7.1]	FGO69_00295	4436.7	5627.6	4993.0	3914.2	5870.7	7573.0	4850.0	7643.6	9465.4

	porD; pyruvate ferredoxin oxidoreductase delta subunit [EC:1.2.7.1]	FGO69_00285	4624.4	5646.0	3629.5	4483.6	6134.1	7601.9	4301.8	7049.1	9495.1
	porG; pyruvate ferredoxin oxidoreductase gamma subunit [EC:1.2.7.1]	FGO69_00280	1338.1	2324.6	1294.9	1123.9	1442.7	2892.1	724.5	1202.2	2344.6
**DLD**	DLD, lpd, pdhD; dihydrolipoamide dehydrogenase [EC:1.8.1.4]	FGO69_05155	278.1	313.1	360.5	386.7	378.7	475.4	376.5	422.0	422.4
**Kor**	korA; 2-oxoglutarate/2-oxoacid ferredoxin oxidoreductase subunit alpha [EC:1.2.7.3, 1.2.7.11]	FGO69_10555	119.2	132.0	188.6	143.0	150.9	153.8	161.7	179.6	183.5
	korC; 2-oxoglutarate ferredoxin oxidoreductase subunit gamma [EC:1.2.7.3]	FGO69_10545	205.1	167.8	278.7	167.3	184.6	208.5	149.7	234.1	246.2
	korB; 2-oxoglutarate/2-oxoacid ferredoxin oxidoreductase subunit beta [EC:1.2.7.3 1.2.7.11]	FGO69_10550	93.1	123.4	140.0	112.2	120.3	150.8	112.1	170.0	177.5
**Fae**	5,6,7,8-tetrahydromethanopterin hydrolyase [EC:4.2.1.147]	FGO69_01010	364.4	290.4	343.5	194.6	214.8	427.7	204.5	181.4	307.2
	bifunctional enzyme Fae/Hps [EC:4.2.1.147, 4.1.2.43]	FGO69_08345	942.6	1371.1	1232.0	894.9	1457.2	1453.6	1127.9	1466.9	1642.4
**Fdh**	fdhA; formate dehydrogenase (NADP+) alpha subunit [EC:1.17.1.10]	FGO69_09900	93.5	84.6	146.2	60.1	81.5	89.4	82.2	91.4	94.5
**Pfl**	pflX; putative pyruvate formate lyase activating enzyme [EC:1.97.1.4]	FGO69_09940	63.8	39.9	83.2	23.2	30.0	55.0	32.6	29.0	41.7
	pflA; pyruvate formate lyase activating enzyme [EC:1.97.1.4]	FGO69_03305	1259.6	931.8	468.7	1580.6	1034.4	702.6	1425.2	1035.3	584.8
**ComA**	comA; phosphosulfolactate synthase [EC:4.4.1.19]	FGO69_10605	139.8	195.3	203.4	130.9	177.4	253.1	114.0	164.2	255.7
		FGO69_10705	140.4	141.7	120.1	115.8	126.0	186.9	110.6	123.1	172.8
**ComB**	comB; 2-phosphosulfolactate phosphatase [EC:3.1.3.71]	FGO69_01195	185.7	321.4	266.2	331.4	446.9	544.8	395.7	339.4	488.6

**ComC**	comC; L-2-hydroxycarboxylate dehydrogenase (NAD+) [EC:1.1.1.337]	FGO69_02530	161.4	189.0	209.9	111.1	168.1	153.7	147.9	173.0	143.8
**ComE**	comE; sulfopyruvate decarboxylase subunit beta [EC:4.1.1.79]	FGO69_02545	252.6	289.1	276.4	251.9	295.8	312.1	338.0	288.0	321.2
	comD; sulfopyruvate decarboxylase subunit alpha [EC:4.1.1.79]	FGO69_02540	166.5	203.7	291.2	184.1	198.4	233.5	259.7	180.1	258.4
**Pps**	Pps; pyruvate, water dikinase [EC:2.7.9.2]	FGO69_09760	243.8	219.6	235.1	188.4	209.6	298.9	256.5	202.8	258.7
**CofH**	cofH; 5-amino-6-(D-ribitylamino)uracil---L-tyrosine 4-hydroxyphenyl transferase [EC:2.5.1.147]	FGO69_06045	94.0	49.7	201.9	83.1	64.9	79.0	101.9	59.1	77.3
		FGO69_07605	60.5	43.8	94.3	38.3	44.0	42.1	59.1	34.2	33.5
**CofG**	cofG; 7,8-didemethyl-8-hydroxy-5-deazariboflavin synthase [EC:4.3.1.32]	FGO69_02480	155.4	118.8	259.7	102.1	133.4	182.8	156.1	122.0	156.1
**CofC**	cofC; 2-phospho-L-lactate guanylyltransferase [EC:2.7.7.68]	FGO69_05810	194.4	260.2	199.9	166.5	259.0	263.7	221.3	290.2	299.0
**CofD**	cofD; LPPG:FO 2-phospho-L-lactate transferase [EC:2.7.8.28]	FGO69_09555	100.5	78.0	151.0	53.7	69.0	105.6	74.1	50.3	84.1
**CofE**	cofE, fbiB; coenzyme F420-0:L-glutamate ligase / coenzyme F420-1:gamma-L-glutamate ligase [EC:6.3.2.31 6.3.2.34]	FGO69_09560	160.0	140.8	245.7	93.6	145.8	189.8	135.8	148.8	186.6

Table S8 | Hydrogenotrophic methanogenesis pathway enzymes and energy conservation. Adapted from Buan, 2018 and Thauer et al., 2008 [1,2].

Reaction	Equation	ΔGº' (kJ/mol)	Enzyme
1	CO_2 + MFR + Fd_{red}^{2-} + $2H^+$ → CHO – MFR + Fd_{ox} + H_2O	0	Formyl-methanofuran dehydrogenase, Fwd
2	CHO–MFR + H_4MPT → CHO – H_4MPT + MFR	–5	Formyl-methanofuran: H_4MPT formyl transferase, Ftr
3	CHO – H_4MPT + H^+ → CH ≡ H_4MPT^+ + H_2O	–5	Methenyl- H_4MPT cyclohydrolase, Mch
4	CH ≡ H_4MPT^+ + $F_{420}H_2$ → CH_2 = H_4MPT + F_{420} + H^+	+6	F_{420}-dependent methylene- H_4MPT dehydrogenase, Mtd
	CH ≡ H_4MPT^+ + H_2 → CH_2 = H_4MPT + H^+	–6	H_2-dependent methylene-H_4MPT dehydrogenase, Hmd
5	CH_2 = H_4MPT + $F_{420}H_2$ → CH_3 – H_4MPT + F_{420}	–6	F_{420}-dependent methylene- H_4MPT reductase, Mer
6	CH_3 – H_4MPT + HS – CoM → CH_3 – S – CoM + H_4MPT	–30	Methyl- H_4MPT:coenzyme M methyltransferase, Mtr
7	CH_3 – S – CoM + H_4MPT + HS – CoB → CH_4 + CoM – S – S – CoB	–30	Methyl-coenzyme M reductase, Mcr
8	H_2 + Fd_{ox} → Fd_{red}^{2-} + $2H^+$	+16	Ferredoxin reducing hydrogenase, Eha/Ehc
9	2 H_2 + CoM – S – S – CoB + Fd_{ox} → HS – CoM + HS – CoB + Fd_{red}^{2-} + 2 H^+	–39	Electron-bifurcating hydrogenase:heterodisulfide reductase complex, Mvh:HdrABC
10	ADP + P_i → ATP + H_2O	–32	ATP synthase
11	$2H^+$ (outside) + $1Na^+$ (inside) → $2H^+$ inside) + $1Na^+$ (outside)	0	Sodium-proton antiporter, MrpA
12	CH_3COOH + CoA – SH + ATP → CH_3 – CoA + AMP + PPi	–6	Acetyl-coA synthetase, ACS
	$4H_2$ + CO_2 → CH_4 + $2H_2O$	–131	Overall hydrogenotrophic methanogenesis reaction

Table S9 | Summary of selected electro-methanogenesis studies in MES with CO_2 as the sole carbon source at the cathode operated at different cathode potentials.

E_{cat} (V vs. SHE)	Cathode	j (A/m^2)	CH_4 ($mmol/m^2/d$)	Ref.
–0.4	GR	0.05	3.5	[3]
–0.5	CC	0.04	0.2	[4]
–0.5	CC	0.05	0.04	[5]
–0.5	CC	3.1	365	[6]
<–0.5	GFB	0.30	200	[7]
–0.55	GF	0.25	4.6	[8]
<–0.6	GF	1.60	205	[9]
–0.7	GF	0.78	38.2	[8]
–0.7	GF	5.9	437	[10]
–0.8	Porous CF	12 – 15	603	[11]
–0.8	GG	0.07	5.1	[12]
–0.8	GG	0.2	15.4	[12]
–0.8	CC	0.5	24	This study
–0.82	GFB	1.6	60.5	[13]
–0.84	GFB	1.42	118.2	[13]
–0.9	CP	0.69 *	400	[14]
–0.95	CF	0.17 *	22.6 *	[15]

CC, carbon cloth; CF, carbon felt; CP, carbon paper; EBC, enriched biocathode; GB, graphite block; GF graphite felt, GFB graphite fiber brush; GG granular graphite; GR, graphite rod. * calculated based on reported data and standard conditions.

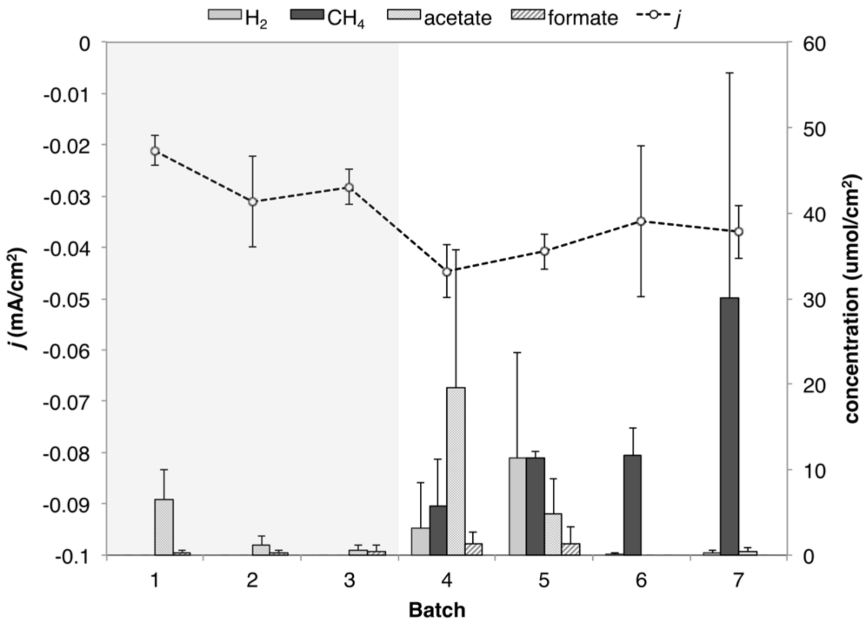

Figure S1 | Average MES performance at –1.0 V vs. Ag/AgCl for the triplicate reactors. The line graph represents the current density *j* on the primary y-axis, while the bar charts represent the measured product concentration (H_2, CH_4, acetate, formate) on the secondary y-axis for each batch. The shaded area indicates the time period when no gas data were available (Batches 1 – 3).

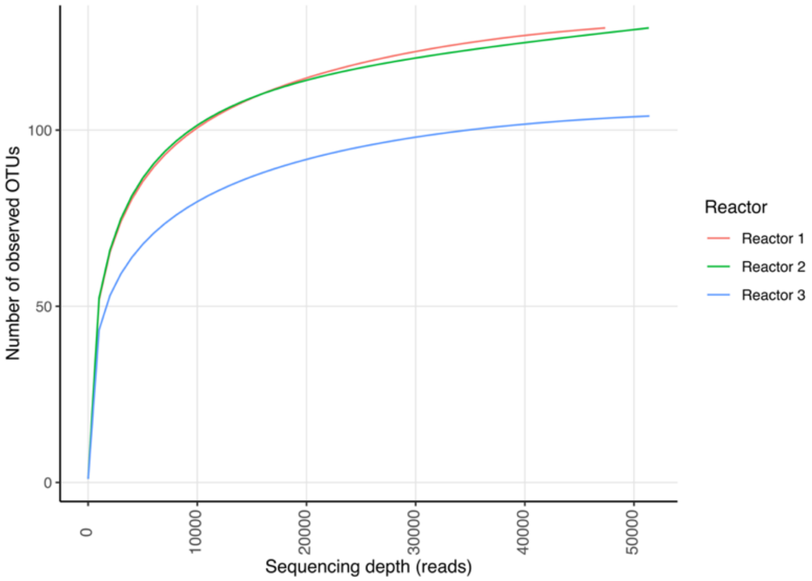

Figure S2 | Rarefaction curve for the three –1.0 V baseline biofilm samples from the triplicate reactors. Samples were rarefied to 47,369 reads in subsequent analyses.

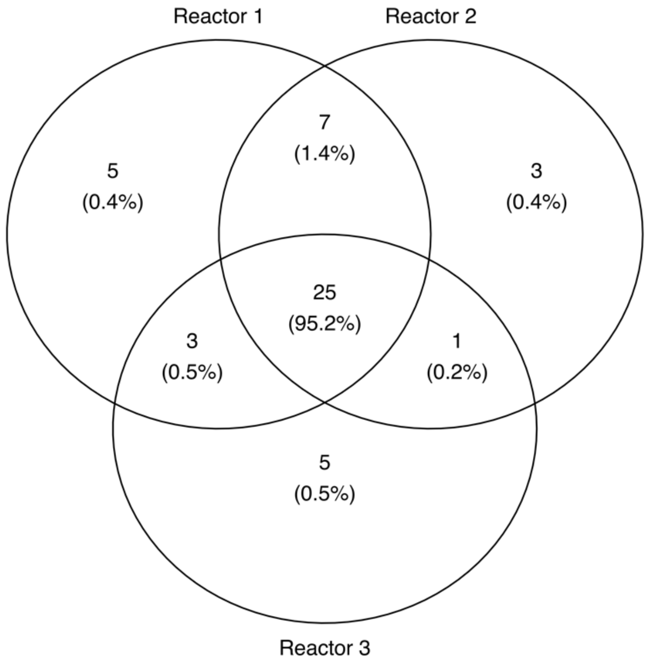

Figure S3| Venn diagram of the distribution of the dominant OTUs (relative abundance ≥0.1%) amongst the three reactors. 25 OTUs out of the 49 dominant OTUs represented the core dominant community, which is defined as OTUs present in all samples at a relative abundance ≥0.1%.

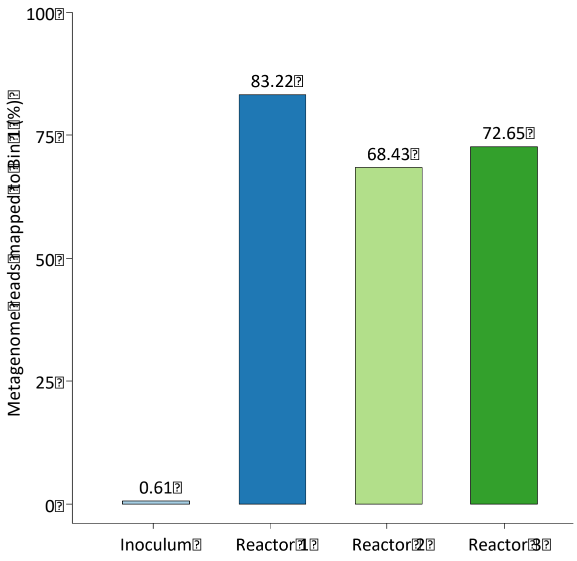

Figure S4 | Abundance of reads mapped to Bin 1 out of the total reads generated in each respective metagenome.

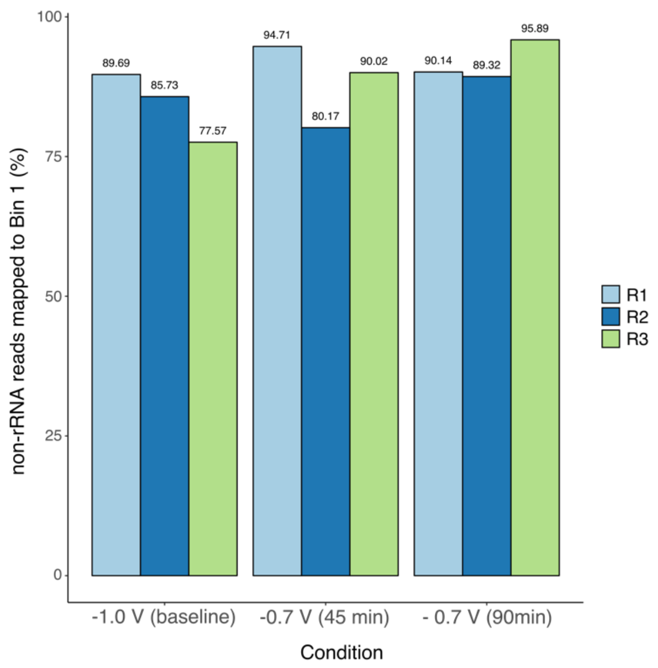

Figure S5 | Abundance of non-rRNA reads (%) mapped to Bin 1 out of the total number of reads generated at each condition for each reactor.

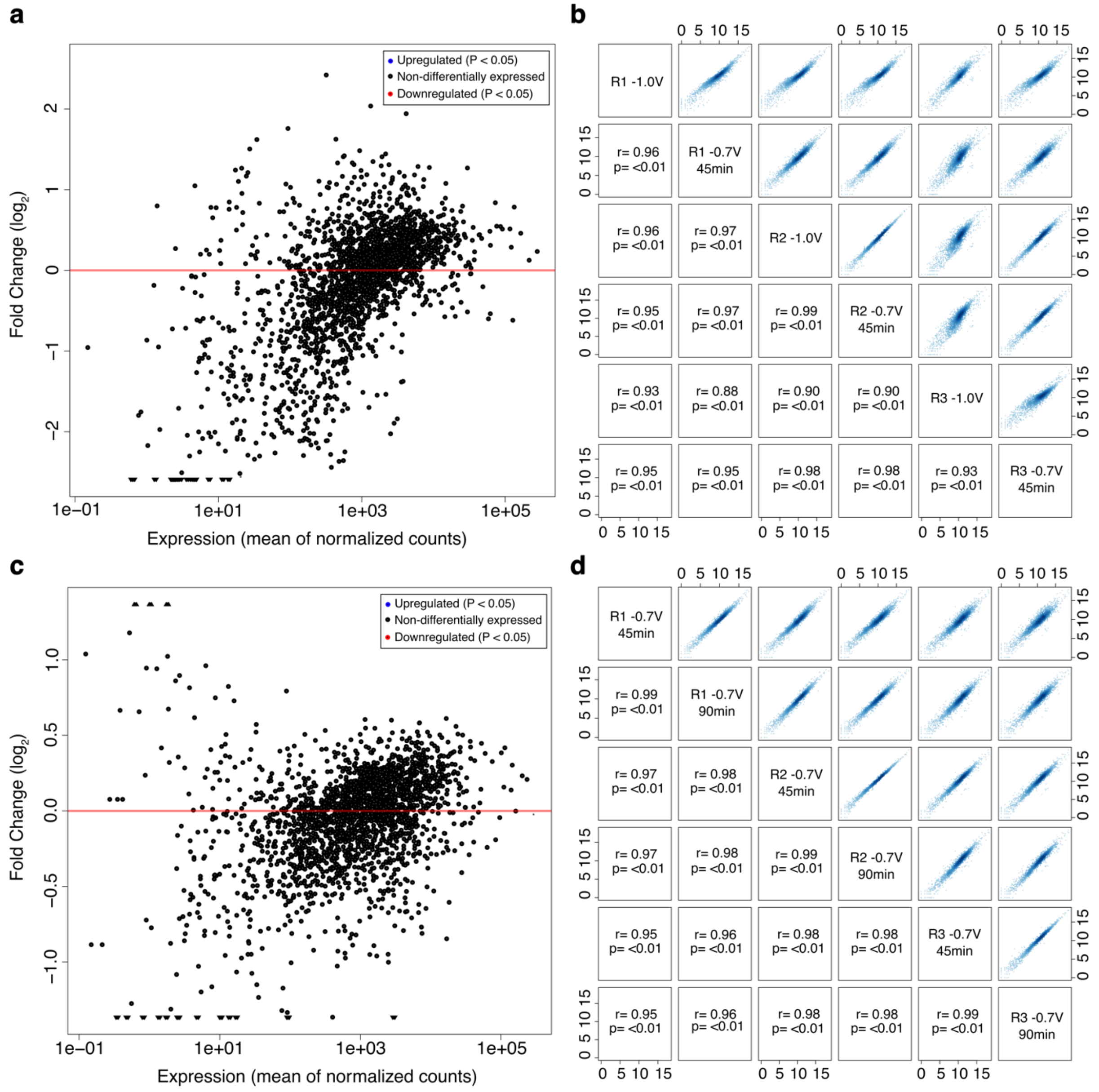

Figure S6 | A differential expression analysis of genes (MA-plots) showing the fold change (log2) as a function of the average expression of each gene. Panel "a" shows the comparison of the expression between –1.0 V vs. –0.7 V after 45 min while Panel "c" shows the comparison between –0.7 V after 45 min vs. after 90 min. Panels "b" and "d" show a pairwise comparison of the number of reads per gene per sample. The counts were normalized to $\log_2$+1 values. Panel "b" shows the normalized counts while Panel "d" shows the corresponding Pearson's correlation coefficient between samples and their *p*-values.

References for Supplementary Information

1. Buan, N. R. Methanogens: pushing the boundaries of biology. *Emerg. Top. Life Sci.* **2**, 629–646 (2018).
2. Thauer, R. K., Kaster, A. K., Seedorf, H., Buckel, W. & Hedderich, R. Methanogenic archaea: Ecologically relevant differences in energy conservation. *Nat. Rev. Microbiol.* **6**, 579–591 (2008).
3. Beese-Vasbender, P. F., Grote, J.-P., Garrelfs, J., Stratmann, M. & Mayrhofer, K. J. J. Selective microbial electrosynthesis of methane by a pure culture of a marine lithoautotrophic archaeon. **102**, 50–55 (2015).
4. Bretschger, O. *et al.* Functional and taxonomic dynamics of an electricity-consuming methane-producing microbial community. *Bioresour. Technol.* **195**, 254–264 (2015).
5. Babanova, S. *et al.* The Effect of Membrane Type on the Performance of Microbial Electrosynthesis Cells for Methane Production. *J. Electrochem. Soc.* **164**, H3015–H3023 (2016).
6. Fu, Q. *et al.* Bioelectrochemical analyses of the development of a thermophilic biocathode catalyzing electromethanogenesis. *Environ. Sci. Technol.* **49**, 1225–1232 (2015).
7. Cheng, S., Xing, D., Call, D. F. & Logan, B. E. Direct Biological Conversion of Electrical Current into Methane by Electromethanogenesis - EST 2009 - Logan.pdf. **43**, 3953–3958 (2009).
8. Van Eerten-Jansen, M. C. A. A., Heijne, A. Ter, Buisman, C. J. N. & Hamelers, H. V. M. Microbial electrolysis cells for production of methane from CO 2: Long-term performance and perspectives. *Int. J. Energy Res.* **36**, 809–819 (2012).
9. Van Eerten-Jansen, M. C. a a *et al.* Microbial community analysis of a methane-producing biocathode in a bioelectrochemical system. *Archaea* **2013**, (2013).
10. van Eerten-Jansen, M. C. a. a. *et al.* Analysis of the mechanisms of bioelectrochemical methane production by mixed cultures. *J. Chem. Technol. Biotechnol.* **31**, n/a-n/a (2014).
11. Dykstra, C. M. & Pavlostathis, S. G. Methanogenic Biocathode Microbial Community Development and the Role of Bacteria. *Environmental Science and Technology* vol. 51 5306–5316 (2017).
12. Batlle-Vilanova, P. *et al.* Deciphering the electron transfer mechanisms for biogas upgrading to biomethane within a mixed culture biocathode. *RSC Adv.* **5**, 52243–52251 (2015).
13. Luo, X. *et al.* Methane production in microbial reverse-electrodialysis methanogenesis cells (MRMCs) using thermolytic solutions. *Environ. Sci. Technol.* **48**, 8911–8918 (2014).
14. Villano, M. *et al.* Bioelectrochemical reduction of CO2 to CH4 via direct and indirect extracellular electron transfer by a hydrogenophilic methanogenic culture. *Bioresour. Technol.* **101**, 3085–3090 (2010).
15. Jiang, Y. *et al.* Bioelectrochemical systems for simultaneously production of methane and acetate from carbon dioxide at relatively high rate. *Int. J. Hydrogen Energy* **38**, 3497–3502 (2013).
